# Greenland Ice Core Record of Last Glacial Dust Sources and Atmospheric Circulation

**DOI:** 10.1029/2022JD036597

**Published:** 2022-08-05

**Authors:** G. Újvári, U. Klötzli, T. Stevens, A. Svensson, P. Ludwig, T. Vennemann, S. Gier, M. Horschinegg, L. Palcsu, D. Hippler, J. Kovács, C. Di Biagio, P. Formenti

**Affiliations:** ^1^ Centre for Astronomy and Earth Sciences Institute for Geological and Geochemical Research Eötvös Loránd Research Network Budapest Hungary; ^2^ CSFK MTA Centre of Excellence Budapest Hungary; ^3^ Department of Lithospheric Research University of Vienna Vienna Austria; ^4^ Department of Earth Sciences Uppsala University Uppsala Sweden; ^5^ Physics of Ice, Climate and Earth Niels Bohr Institute University of Copenhagen Copenhagen Denmark; ^6^ Institute for Meteorology and Climate Research Karlsruhe Institute of Technology Karlsruhe Germany; ^7^ Institute of Earth Surface Dynamics University of Lausanne Lausanne Switzerland; ^8^ Department of Geology University of Vienna Vienna Austria; ^9^ Isotope Climatology and Environmental Research Centre Institute for Nuclear Research Debrecen Hungary; ^10^ Institute of Applied Geosciences Graz University of Technology Graz Austria; ^11^ Environmental Analytical and Geoanalytical Research Group Szentágothai Research Centre University of Pécs Pécs Hungary; ^12^ Institute of Geography and Earth Sciences University of Pécs Pécs Hungary; ^13^ Université de Paris Cité and University Paris Est Creteil CNRS LISA Paris France

**Keywords:** NGRIP ice core, mineral dust, aerosol, isotopic fingerprinting, Greenland

## Abstract

Abrupt and large‐scale climate changes have occurred repeatedly and within decades during the last glaciation. These events, where dramatic warming occurs over decades, are well represented in both Greenland ice core mineral dust and temperature records, suggesting a causal link. However, the feedbacks between atmospheric dust and climate change during these Dansgaard–Oeschger events are poorly known and the processes driving changes in atmospheric dust emission and transport remain elusive. Constraining dust provenance is key to resolving these gaps. Here, we present a multi‐technique analysis of Greenland dust provenance using novel and established, source diagnostic isotopic tracers as well as results from a regional climate model including dust cycle simulations. We show that the existing dominant model for the provenance of Greenland dust as sourced from combined East Asian dust and Pacific volcanics is not supported. Rather, our clay mineralogical and Hf–Sr–Nd and D/H isotopic analyses from last glacial Greenland dust and an extensive range of Northern Hemisphere potential dust sources reveal three most likely scenarios (in order of probability): direct dust sourcing from the Taklimakan Desert in western China, direct sourcing from European glacial sources, or a mix of dust originating from Europe and North Africa. Furthermore, our regional climate modeling demonstrates the plausibility of European or mixed European/North African sources for the first time. We suggest that the origin of dust to Greenland is potentially more complex than previously recognized, demonstrating more uncertainty in our understanding dust climate feedbacks during abrupt events than previously understood.

## Key Points

- Hf–Nd isotope data demonstrate that Central Greenland last glacial dust is not sourced from mixed SE Asian and circum‐Pacific volcanic dust
- Most likely sources are the Taklimakan in SE Asia, European and/or North African glacial dust deposits based on Hf–Sr–Nd and D/H isotopes
- Model simulations show that sourcing of North Greenland Ice Core Project ice core dust from Europe is plausible, the addition of North African dust cannot be excluded

## 1 Introduction

Abrupt climate changes have occurred repeatedly and within decades in the recent geological past (Alley et al., 2003), including the last glaciation. These changes were centered on the North Atlantic, where vigorous wind systems encountered the southernmost extension of sea ice and oceanic currents with connections to the deep ocean via convection (Li & Born, 2019; Lynch‐Stieglitz et al., 2007). There is growing concern that recent CO_2_ forcing and warming will lead to a global cascade of rapid regime shifts in the near future (Lenton et al., 2019), resulting in abrupt climate changes, which are difficult to model and predict, but would be a global threat to civilization. The reason for this lack of ability to predict tipping points is due to the limited understanding of feedbacks in the climate system during abrupt events. In particular, the role of atmospheric dust, which has both direct and indirect influence on regional and global climate through diverse physical and biogeochemical processes and feedbacks (Mahowald, 2011; Sokolik & Toon, 1996; Tegen et al., 1996), is still poorly known.

**Figure 1:**
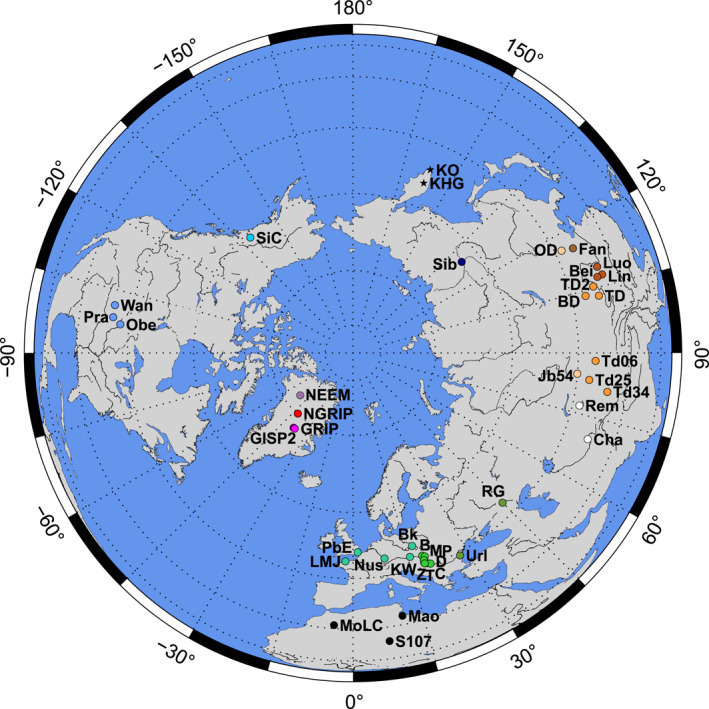
Locations of the North Greenland Ice Core Project (NGRIP) and other central Greenland ice cores and potential source area samples across the Northern Hemisphere. Sites displayed on map are as follows (codes also listed in Dataset S1 and S2). Greenland: NGRIP – North Greenland Ice Core Project ice core, GRIP ‐ Greenland Ice Core Project ice core, GISP2 – Greenland Ice Sheet Project 2 ice core, NEEM ‐ North Greenland Eemian Ice Drilling. North America: SiC – Silver Creek (Yukon, Canada), Obe – Obert (Nebraska, USA), Pra – Prairie Lake (Nebraska, USA), Wan – Wauneta (Nebraska, USA). European sites are abbreviated, where necessary (with original codes given in parenthesis, and in Dataset S2): B – Basaharc (Bh, Hungary), Bk – Bialy Kosciol (Poland), C – Crvenka (Crv, Serbia), D – Dunaszekcső (Dsz, Hungary), KW – Krems‐Wachtberg (KW‐GH9, Austria), LMJ – La Motte (Jersey, Channel Islands), M – Mende (Me, Hungary), Nus – Nussloch (Germany), P – Paks (Pa, Hungary), PbE – Pegwell Bay (UK), RG – Raigorod (Lower Volga, Russia), T – Titel core (Tic‐1A, Serbia), Url – Urluia (Romania), Z – Zmajevac (Zm, Croatia). North Africa: Mao – Maouna (Tunisia), MoLC – off‐road site (Erg Chebbi, Morocco), S107 – off‐road site (Ahaggar Mts., Algeria). Central Asia: Cha – Chasmanigar (Tajikistan), Rem – Remizovka (Kazakhstan). Northeast Asia: KHG – Khangar volcano (Kamchatka, Russia), KO – Kurile lake (Kamchatka, Russia), Sib – Tumara valley (Siberia, Russia). Southeast Asia: BD – road site (Badain Jaran desert, China) Bei – Beiguoyuan (Chinese Loess Plateau (CLP), China), Fan – Fanshan (around Beijing, China), Jb54 – dune site (Junggar basin, China), Lin – Lingtai (CLP, China), Luo – Luochuan (CLP, China), OD – road site (Otin Dag desert, China), TD/TD2 – road sites (Tengger desert, China), Td06/Td25/Td34 – overbank, stream (fan) and loess sites (Taklimakan desert, China). Regional color coding corresponds with those in Figures 2, 3, 4.

Abrupt changes in climate of the Northern Hemisphere (NH) over the last glaciation are well recorded in Greenland ice. High‐resolution ice core oxygen isotope records reveal that the cold glacial climate was interrupted by numerous rapid shifts to warmer interstadial conditions, called Dansgaard–Oeschger (D–O) events, over the last glaciation (Bond et al., 1993; Dansgaard et al., 1993). Air temperatures rose by 5–16°C within decades during D–O events in Greenland (Huber et al., 2006; Kindler et al., 2014), followed by a much slower drop in temperature back to stadial conditions. This D–O type climate variability is coupled with dust concentration and particle size variations, with rapid decreases in Ca^2+^ ions (a dust concentration proxy) at the onset of interstadials and slower increases following their end (Fuhrer et al., 1999). Dust particle concentrations in the Greenland Ice Core Project (GRIP) and North Greenland Ice Core Project (NGRIP, Figure 1) ice cores were found to be higher by a factor of 100 during the Last Glacial Maximum (LGM: 26–19 ka, Clark et al., 2009) than the Preboreal warm period and typically a factor of 8 higher at the sharp transitions of D–O events, with modes of particle volume size distributions being systematically coarser over cold periods (Ruth et al., 2003; Steffensen, 1997). These abrupt variations in dust characteristics were variously accounted for by changes in transit and atmospheric residence time, transport distance, extent and intensity of the polar atmospheric cell, dust source strength, aridity and storminess (Fischer et al., 2007; Hansson, 1994; Mayewski et al., 1994; Ruth et al., 2003; Schüpbach et al., 2018; Steffensen, 1997), nonetheless the precise causal mechanisms of these changes remain elusive. Comparison of *δ* ^18^ *O*_ice_ and dust (mass, Ca^2+^) records of the NGRIP ice core demonstrates that the average lag of dust versus *δ* ^18^ *O*_ice_ is 1 ± 8 years for all D–O inceptions (Ruth et al., 2007). This synchrony of dust concentration and *δ* ^18^ *O*_ice_ changes has recently been confirmed by higher resolution analyses of NGRIP and NEEM ice core data (Capron et al., 2021; Erhardt et al., 2019), indicating that the climate of continental dust sources and Greenland must have been coupled. However, the mechanisms of this coupled response over wide areas of the NH remain poorly constrained. Resolving the source(s) of Greenland ice core dust is a key step in resolving the uncertainty over this coupling, as it allows the environmental controls on dust emission to be constrained, as well as provides insight into major dust transport pathways and atmospheric circulation patterns during abrupt climate events of the Last Glacial Period. This facilitates a better understanding of feedbacks between dust and climate at decadal to millennial timescales and the role of different atmospheric patterns in dispersing dust over the NH.

Early studies of the clay mineralogy and Sr‐Nd‐Pb isotopic compositions of dust aerosols in the Greenland Ice Sheet Project 2 (GISP2) and GRIP ice cores suggested derivation from the SE Asian deserts (e.g., Gobi, Taklimakan) and Chinese Loess Plateau (CLP) over the LGM, with additions of circum‐Pacific volcanic material in proportions of 10%–25% to account for the Sr isotope compositions of ice core dust (Biscaye et al., 1997; Svensson et al., 2000). Later work partly supported this SE Asian origin (Burton et al., 2007; Han et al., 2018; Meyer et al., 2017), and also for preindustrial aerosols in the Dye‐3 ice core (Lupker et al., 2010) or modern dust collected in snow pits at the NGRIP site (Bory, Biscaye, & Grousset, 2003). However, despite the wide acceptance of this model, the sources of dust have not as yet been unequivocally constrained and other potential dust sources to Greenland, including Africa (Han et al., 2018; Meyer et al., 2017) and central Europe (Újvári et al., 2015), and admixtures of dust from sources other than SE Asia (Lupker et al., 2010; Meyer et al., 2017), were also proposed based on expanded datasets and currently are equally supported by available data. Studies of preindustrial/Holocene aerosols recovered from ice cores at lower elevation sites, located closer to the edge of the ice cap (Hans Tausen, Renland, RECAP) demonstrate dust derivation from proximal sources (Bory, Biscaye, Piotrowski, & Steffensen, 2003), including central East Greenland (Simonsen et al., 2019).

Model simulations of the LGM dust cycle are likewise diverse in simulated potential dust sources to central Greenland. SE Asia is one of the major LGM dust emission centers and aerosol sources for central Greenland ice in numerous models (De Angelis et al., 1997; Lunt & Valdes, 2001; Mahowald et al., 1999, 2006, 2011; Reader et al., 1999; Werner et al., 2002), while glaciogenic/non‐glaciogenic sources in the continental USA, Alaska and Siberia are identified as additional potential contributors (Lambert et al., 2015; Mahowald et al., 2006). An active dust cycle in Europe can also be identified in regional simulations (Ludwig et al., 2021; Schaffernicht et al., 2020). Over the LGM, the Sahara Desert was the most active and largest dust source (Albani et al., 2018; Takemura et al., 2009; Werner et al., 2002) and the shift of the Hadley cell circulation outer boundary toward the equator may have enhanced desert source areas by factors of 2–3 during the last glacial, allowing meridional dust transport by mid‐latitude storm systems to polar regions (Chylek et al., 2001).

As such, despite the prevalence of the SE Asian desert/loess model for dust sources to Greenland, available dust provenance data and computer simulations provide a range of plausible scenarios. Such ambiguities mean we have limited knowledge of wider glacial dust loading and present a major impediment to understanding what specific areas were emitting dust during abrupt climate change events, the cause of these emissions, and the consequences for global climate. To gain deeper insights into this inherently complex problem, we present new Sr–Nd–Hf and D/H isotope compositional data from early LGM (25.64–24.85 ka) dust samples of the NGRIP ice core and new clay mineralogy and isotopic data from the fine (<5 and <2 μm) fractions of a large range of potential source area (PSA) samples collected from major last glacial dust hotspots over the NH (Figure 1), combined with new regional model simulations of LGM dust transport over Europe. Since Sr isotope compositions strongly suffer from grain size and pre‐treatment effects and Nd isotopes are not source diagnostic for Greenland ice core dust (Újvári et al., 2015), two additional, novel dust tracers are applied here; Hf and H isotopes. To our knowledge, hafnium isotope ratios have only once been utilized to trace sources of mineral dust from preindustrial segments in the Dye‐3 ice core (Lupker et al., 2010), potentially due to analytical difficulties, yet are likely highly source diagnostic (Újvári et al., 2015, 2018). A recently established methodology developed for the combined Hf–Sr–Nd isotope measurements of low mass dust samples (2–10 mg; Újvári et al., 2021) has now made it possible to analyze the Hf isotope compositions of a relatively high number of PSA samples, together with ice core dust. In addition, the D/H isotope composition of clay structural water (*δD*_csw_, i.e., that of hydroxyl groups of clays; Savin & Epstein, 1970) of size separated dust samples, has been analyzed for the first time in ice core dust provenance research. In general, the D/H isotope composition of naturally occurring clay minerals (kaolinite, montmorillonite, etc.) are used to reconstruct conditions under which the minerals are formed, and thus to better understand paleoclimate and pedogenetic processes (Savin & Hsieh, 1998; Sheppard & Gilg, 1996). Here, *δD*_csw_, which is a combined signal of a mixture of clay minerals present in the dust separates, is simply used as a source fingerprint and not for making inferences about the paleoclimate. This, we will show, is feasible given that the PSAs form distinct groups in terms of *δD*_csw_, allowing further constraints on the origin of ice core dust. Finally, we present regional model simulations results of the LGM dust cycle for Europe (including the northernmost part of Africa) with the WRF‐Chem model to investigate if dust emitted from these areas may have contributed to dust deposition on central parts of the Greenland Ice Sheet, as this potential pathway has not been simulated up until today.

## 2 Materials and Methods

### 2.1 Studied Samples and Pretreatments

Four ice core samples were selected for this study from the NGRIP ice core, Greenland (Figure 1), corresponding to one of the periods with the highest dust accumulation of the LGM. When selecting the samples, we focused on the GS‐3 stadial period (Figure S1 in Supporting Information S1), which is characterized by the highest dust concentrations (4–8 mg dust/kg ice; Ruth et al., 2003) and did not investigate interstadials with much lower concentrations (0.3–0.7 mg dust/kg ice) as at least 2–3 mg dust was required for the combined Sr–Nd–Hf and H isotope analyses. The sample bags 3,327, 3,326, 3,323 and 3,306–3,307 span depths of 1829.85–1818.30 m and cover the age intervals of 25,640–25604, 25,604–25567, 25,491–25453 and 24,924–24859 years GICC05 b2k (Text S1, Figure S1 in Supporting Information S1). Impurities were obtained by melting about 0.6 kg ice each (except for the combined bags 3,306–3,307: about 1 kg) at room temperature, after removing the outer ca. 3–5 mm of ice from all sides with pre‐cleaned PFA chisel. Melted ice samples were collected in acid‐cleaned Savillex PFA beakers and evaporated to incipient dryness at 50°C in clean laboratory environment in Vienna. Subsequently, the impurities were treated with weak (0.5 mol/L) acetic acid for 1 hr to remove carbonates and sea salt aerosols of marine origin, following Svensson et al. (2000). Both the leachates and remaining aluminosilicate fractions were analyzed for isotopic compositions.

PSA samples include modern soil, desert/dune sand, overbank, stream/fan and lake deposits and late Quaternary loess deposits, listed in Datasets S1 and S2 (also in Újvári, 2021). Loess sediments were collected from luminescence‐ or radiocarbon‐dated sequences and most of them represent LGM or last glacial (L1) dust of the respective regions. Two pumice fall deposits of Holocene age were also investigated from Kamchatka, one of them (KHG) identified in Greenland ice cores. Long‐range atmospheric transport fractionates dust and only the finest fractions (typically <10 or <5 μm) can reach Greenland from distal continental sources, as evidenced by the particle size distribution of the NGRIP ice core (Ruth et al., 2003), which is dominated by <10 μm particles (Figure S2b in Supporting Information S1). To minimize grain size and mineralogy effects on the isotopic compositions and ensure that the same particle size fractions of PSA and ice core samples are compared, the PSA samples were size separated using 5 μm hydrophobic Mitex filter and/or wet sedimentation (Stokes law) (Text S2, Figure S2 in Supporting Information S1). The <5 and <2 μm size separates were subsequently leached in 0.5 mol/L acetic acid for 1 hr and washed in 30% H_2_O_2_ to remove secondary carbonates and organic matter. The residues (aluminosilicate fractions) were washed with Milli‐Q water and dried (50°C) for subsequent XRD or isotopic analyses.

### 2.2 Powder X‐Ray Diffraction

For clay mineral analyses samples were disaggregated with diluted H_2_O_2_ to remove organic matter and the slurry was subsequently prepared for XRD analyses as described in detail in Moore and Reynolds (1997). The prepared clay samples were analyzed with a Panalytical PW 3040/60X’Pert PRO diffractometer (CuKα radiation, 40 kV, 40 mA, step size 0.0167, 5 s per step). The X‐ray patterns were interpreted following Moore and Reynolds (1997). Smectite is identified by a broad peak at about 14 Å with Mg‐saturation, which shifts to about 12 Å with K‐saturation and collapses to 10 Å after heating to 550°C. Saturation of the K‐saturated sample with ethylene glycol expands smectite again to 17 Å, and the Mg‐saturated sample with glycerol expands to 18 Å. Chlorite was identified by peaks at 14, 7, 4.7 and 3.53 Å, which retain their position during treatments. Illite peaks at 10, 5 and 3.3 Å likewise keep their positions. Kaolinite peaks at 7 and 3.57 Å no longer appear after heating to 550°C. Mg‐saturated vermiculite has a strong peak at 14 Å, and with K saturation it shifts to 10 Å. Ethylene glycol saturation of the K‐saturated sample or glycerol‐saturation of the Mg‐saturated sample do not lead to changes in positions. The clay mineral compositions of samples were quantified after Biscaye (1965), as also described in Svensson et al. (2000). The peak areas of clay minerals in the Mg‐ and glycerol‐saturated X‐ray patterns were determined using the Panalytical X'Pert Highscore plus software.

### 2.3 Hafnium, Neodymium and Strontium Isotope Analyses

All chemical separations in this study were performed in PicoTrace class 100 clean rooms at the Department of Lithospheric Research, University of Vienna, Austria. Details on chemicals, lab wares and columns used in this study, as well as the ion‐exchange chemistry of the elemental separations and purifications can be found in Újvári et al. (2021). Pretreated NGRIP dust samples and size separates of PSA samples were digested in a convection oven at 230°C using ammonium bifluoride (ABF, NH_4_HF_2_), and subsequently by concentrated HNO_3_ and HCl (Újvári et al., 2021). Total chemistry blanks were determined to be 0.5 ng for Sr, 0.2 ng for Nd and <25 pg for Hf, all negligible in the present application. Performance of the ammonium‐bifluoride method together with the column chemistry setup was thoroughly tested and demonstrated using five USGS geological reference materials, including AGV‐2, BCR‐2, GSP‐2, RGM‐2 and STM‐2 (Újvári et al., 2021).

Mass spectrometry of Sr and Nd was performed at the Department of Lithospheric Research, University of Vienna, Austria using a Thermo‐Finnigan Triton TI multi‐collector TIMS instrument in static mode. Pure element fractions were analyzed using a Re double filament assembly. A mean ^87^Sr/^86^Sr ratio of 0.710259 ± 0.000002 (2SE, *n* = 20) was measured for NBS987 (ref. value: ^87^Sr/^86^Sr = 0.710248; Faure, 2001) and a mean ^143^Nd/^144^Nd ratio of 0.511843 ± 0.000002 (2SE, *n* = 31) for the La Jolla (ref. value: ^143^Nd/^144^Nd = 0.511858; Lugmair & Carlson, 1978) isotope standards during the analysis periods. Mass fractionation during measurement was corrected for ^86^Sr/^88^Sr = 0.1194, and ^146^Nd/^144^Nd = 0.721903, respectively. The measured ^87^Sr/^86^Sr and ^143^Nd/^144^Nd isotope ratios of NGRIP dust and PSA samples were finally normalized to the reference isotope ratios of 0.710248 and 0.511858 of the NBS987 and La Jolla isotope standards. Uncertainties of isotopic ratios represent 2 standard errors of the mean (2SE).

Hafnium isotope analyses were done on a Thermo Neptune Plus MC‐ICP‐MS equipped with an Aridus three desolvation nebulizer (flow rates of 100 μl/min) in the laboratory of the Institute of Nuclear Research, Debrecen, Hungary. A Faraday gain calibration was made prior to instrument tuning. Masses of ^172^Yb, ^174^Hf and ^175^Lu were measured using 10^13^ Ω, while others (^176^Hf, ^177^Hf, ^178^Hf, ^179^Hf, and ^180^Hf) using 10^11^ Ω amplifiers. Gain calibration protocol details and Faraday cup configurations are given in Újvári et al. (2021). An exponential mass bias correction for Hf (*β*Hf) is applied using ^179^Hf/^177^Hf = 0.7325 (Blichert‐Toft et al., 1997; Chu et al., 2002; Griffin et al., 2006; Patchett, 1983). ^176^Lu and ^176^Yb interference corrections on ^176^Hf were made using recommended values of ^176^Lu/^175^Lu = 0.026549 and ^176^Yb/^172^Yb = 0.58862 (Chu et al., 2002), with mass bias factors *β*Yb = *β*Lu = *β*Hf. Interference corrected Hf isotope ratios and reported errors represent the mean and 2 standard error of the mean (2SE) values. Repeat analyses of the JMC‐475 Hf isotope standard (10 ppb solutions) yielded mean ^176^Hf/^177^Hf isotopic ratios of 0.282139 ± 0.000035 (2SD, *n* = 29), 0.282141 ± 0.000019 (2SD, *n* = 4) and 0.282152 ± 0.000011 (2SD, *n* = 2) for the three analytical sessions in 2020 versus the accepted value of 0.282163 ± 0.000009 (Blichert‐Toft et al., 1997). The measured ^176^Hf/^177^Hf isotopic ratios were normalized to the accepted ^176^Hf/^177^Hf isotopic ratio of JMC‐475.

Nd and Hf isotopic ratios are also reported as *ε*Nd(0) = ((^143^Nd/^144^Nd_sample_/^143^Nd/^144^Nd_CHUR_) −1) × 10^4^ and *ε*Hf(0) = ((^176^Hf/^177^Hf_sample_/^176^Hf/^177^Hf_CHUR_) −1) × 10^4^ in this study using the present‐day chondritic uniform reservoir (CHUR) values of 0.512630 ± 0.000011 and 0.282785 ± 0.000011 (Bouvier et al., 2008). Uncertainties of *ε*Nd(0) and *ε*Hf(0) values were propagated as $\sqrt{{\left(\left(\partial \varepsilon /\partial x\right)\sigma_{x}\right)}^{2}+{\left(\left(\partial \varepsilon /\partial y\right)\sigma_{y}\right)}^{2}}=\sqrt{{\left(\left(1/y\right)10000\sigma_{x}\right)}^{2}+{\left(\left(-x/y^{2}\right)10000\sigma_{y}\right)}^{2}}$, where *ε* = *ε*Nd(0) or *ε*Hf(0), *x* = ^143^Nd/^144^Nd_sample_ or ^176^Hf/^177^Hf_sample_, *y* = ^143^Nd/^144^Nd_CHUR_ or ^176^Hf/^177^Hf_CHUR_, *σ*_*x*_ and *σ*_*y*_ are uncertainties of *x* and *y* (Újvári et al., 2018). Further information on reproducibility of Sr–Nd–Hf isotope measurements can be found in Text S3 and Figures S3‐S4 of Supporting Information S1.

### 2.4 Hydrogen Isotope Analysis of Clay Structural Water

Measurements of the water content and D/H (^2^H/^1^H) isotope compositions of bulk NGRIP dust, and fine fractions (<5 and <2 μm) of PSA samples were made using high‐temperature (1,450°C) reduction methods with He‐carrier gas and TC‐EA attached to a gas chromatographic column and Thermo‐Finnigan Delta Plus XL mass spectrometer (Bauer & Vennemann, 2014; Sharp et al., 2001). Sample mass ranged from 0.3 to 2.3 mg. To remove adsorbed surface and interlayer water, fine‐grained clay separates were dehydrated in a vacuum line at 250°C for 3 hr and subsequently isolated in small break seal tubes up until analysis with a zero‐blank sample tray on the TC‐EA, allowing for the isotopic analysis of structurally bound water in clays only (Bauer & Vennemann, 2014). Testing with clay standards demonstrated that the TC/EA method provides accurate D/H isotopic compositions and has comparable precision and reproducibility to the Zn‐based method (Bauer & Vennemann, 2014). The results are given in the standard *δ*‐notation (*δ*D), expressed relative to V‐SMOW in permil (‰). The precision of the in‐house kaolinite and G1 biotite standards for hydrogen isotope analyses is better than ±2 ‰. All values were normalized using values of −125 ‰ for the K‐17 kaolinite standard and −65‰ for NBS‐30. Additional information on D/H isotope measurements can be found in Text S4, Figures S5 and S6 of Supporting Information S1.

### 2.5 Dust Emission and Transport Simulations

Simulations of the LGM dust cycle over Europe at high spatial (50 km horizontal grid spacing) and temporal resolution (6 hourly output interval) were performed using the Weather and Research Forecast model with chemistry (WRF‐Chem; version 4.1.2, Skamarock et al., 2019). In WRF‐Chem, the GOCART (Chin et al., 2000; Ginoux et al., 2001) aerosol physics have been implemented by including algorithms for dust emissions, transport and deposition. WRF‐Chem has been used in dust‐only mode. Size resolved dust emissions (five dust bins: 0–2, 2–3.6, 3.6–6, 6–12 and 12–20 μm) were simulated based on the University of Cologne dust emission scheme (Shao, 2004). The dust flux into the atmosphere is triggered by saltation bombardment and aggregate disintegration, depending on the soil texture. Dust emission is then proportional to the friction velocity and the simulations are run in dust‐only mode, implying that only emission, transport, and deposition is considered, without any radiative and/or cloud feedbacks. Additionally, we used the polar WRF extension (version 4.1.1, Hines et al., 2015) to account for an optimized surface energy balance and heat transfer for the Noah land surface model over sea ice and permanent ice surfaces and to update the sea ice concentration during the simulation. The potential dust source areas were determined following the approach by Ginoux et al. (2001). Other physical parametrizations applied in this study are summarized in Table S1 of Supporting Information S1 (Text S5 in Supporting Information S1). The WRF‐Chem model has been successfully used in this configuration to simulate different aspects of the regional LGM climate and dust cycle over Europe (Ludwig et al., 2017, 2021; Schaffernicht et al., 2020).

The WRF model is forced by initial and 6‐hourly lateral boundary conditions, including time‐varying sea‐ice and SST, from global LGM simulation (1.875° × 1.875° horizontal grid spacing in longitude/latitude) by the Max‐Planck‐Institute of Meteorology earth system model (MPI‐ESM‐P, Jungclaus et al., 2012), performed during the third phase of the Paleomodel Intercomparison Project (PMIP3, Braconnot et al., 2012). An overview of the capabilities of the MPI to simulate the general LGM climate conditions and atmospheric circulation in comparison with other global climate models is given in for example, Ludwig et al. (2016) and Kageyama et al. (2021). The MPI‐ESM simulations are very close to the mean of the PMIP3 simulations in terms of temperature and precipitation (Figures 1e and 7e in Kageyama et al., 2021), so the MPI‐ESM‐P cannot be considered as an outlier either at the high or low end of the model spread. In comparison with proxy data, the MPI‐ESM‐P model is in good agreement with the proxy reconstructions for temperature and outperforms some other PMIP3 models, which show larger deviations from proxy data (Figures 10b, 10d and 11 in Kageyama et al., 2021).

The dust in the WRF simulations is only sourced within the regional domain since the MPI‐ESM‐P does not provide any dust data in its standard PMIP3 setup and thus no tracers can be passed at the lateral boundaries. This makes the results much easier to interpret, as the dust that arrives at Greenland can only be attributed to European/North African sources.

Several modifications have been introduced to the WRF model to account for the LGM boundary conditions. These included the implementation of an orbital routine accounting for changes in orbital parameters (Prömmel et al., 2013) and modifications of the vegetation cover and land use (CLIMAP Project Members, 1984; for implementation see Ludwig et al., 2017). Further corrections were related to ice sheets, land‐sea‐mask and trace gas concentration changes based on PMIP3 guidelines for the 21k experiment for consistency with the MPI‐ESM‐P forcing data (https://pmip3.lsce.ipsl.fr/) and the alpine ice shield (Seguinot et al., 2018). An overview of specific model settings is given in Table S2 of Supporting Information S1. The added value of regional paleoclimate model simulations from the WRF model in comparison with the outcome of the global MPI‐ESM‐P model have been described for example, in Ludwig et al. (2017, 2019). Furthermore, Pinto and Ludwig (2020) analyzed the statistics and characteristics of cyclones based on MPI‐ESM‐P driven WRF simulations. The WRF LGM dust simulation for this study comprises 32 years, where the first 2 years have been excluded from the analysis to account for a sufficient model spin‐up time. The horizontal grid spacing is 50 km over the model domain, which included the northern Atlantic Ocean and Europe, with the British and Fennoscandinavian ice sheets (Figure 5). The vertical grid consists of 35 levels up to 50 hPa (∼20 km height) to cover the whole troposphere. The vertical levels are irregularly distributed with height, with the highest vertical resolution in the boundary layer. The time step is set to 180 s for the simulation.

**Figure 2:**
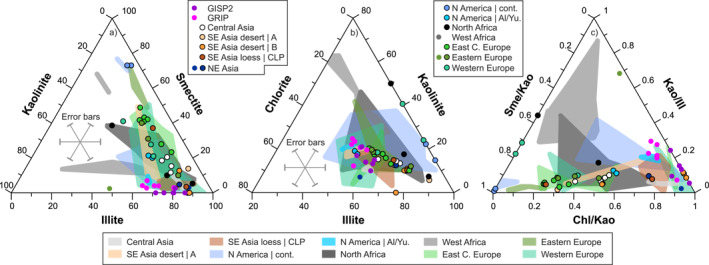
Ternary clay mineralogy diagrams of Greenland Ice Sheet Project 2 (GISP2)/Greenland Ice Core Project (GRIP) ice core dust and potential source areas in the (a) illite‐smectite‐kaolinite, (b) illite‐kaolinite‐chlorite and (c) chlorite/kaolinite‐kaolinite/illite‐smectite/kaolinite space. Note that the XRD data are normalized to 100 percent in each ternary plot. Dots without rims and fields in the background indicate published literature data (<2 μm fractions), while dots with rims denote newly acquired XRD data obtained in this study. Geographic regions represented by only one datum (e.g., NE Asia) is not defined by fields, but a rimless dot. Error bars on panels a) and b) represent a general 10 wt% uncertainty of XRD determination.

**Figure 3:**
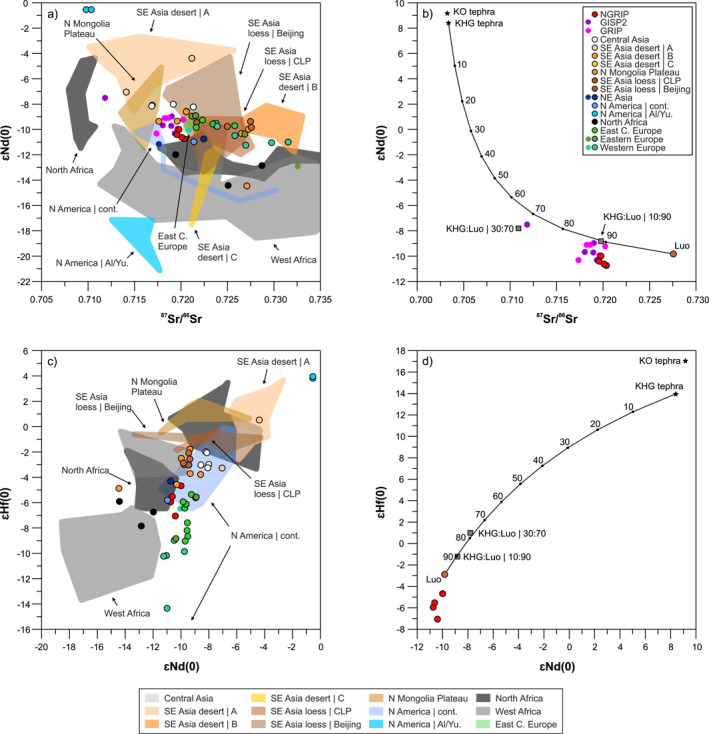
Sr‐Nd (a) and Nd‐Hf (c) isotope compositions of central Greenland last glacial dust and potential source area samples with mixture models in the Sr‐Nd (b) and Nd‐Hf (d) isotope space. Fields and rimless dots indicate literature data (list of data sources in Dataset S2), while dots with rims denote new isotopic data obtained in this study. End‐members displayed on panels (b and d) are loess from Luochuan (Luo, CLP, China) and pumice fall deposit of the Khangar volcano (KHG, Kamchatka, Russia). Mixing lines are calculated with the equations given in Faure and Mensing (2005) and using elemental concentrations of Luo and KHG determined by ICP‐MS and shown in Dataset S2. Measured Sr‐Nd‐Hf isotope compositions of artificial mixtures of KHG and Luo (proportions of 10%–90% and 30%–70%) are displayed in panel (b and d).

**Figure 4:**
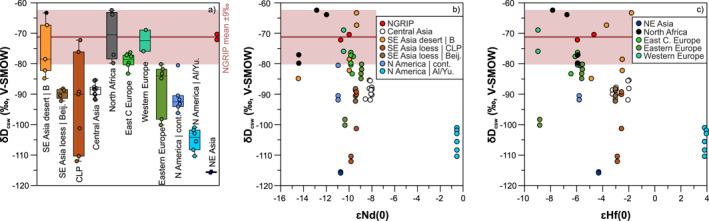
Box/scatter‐(a) and bi‐plots (b–c) of hydrogen isotope compositions of the clay structural water (*δD*_csw_) of North Greenland Ice Core Project (NGRIP) last glacial dust and potential source area (PSA) samples as a function of Nd‐Hf isotope compositions. The NGRIP mean *δD*_csw_ value is −71‰ with a ±9‰ band derived from the repeatability of 2–5 μm separates of PSA samples (see Text S4 and Figure S6 in Supporting Information S1). The *δD*_csw_ of both 2 and 5 μm separates are included in boxes of panel (a) and can be found in Dataset S2. In panel (b–c) all hydrogen isotope data are displayed as measured on both 2 and 5 μm separates, where corresponding Nd and Hf isotope ratios were available from the 5 μm fractions (Dataset S2).

**Figure 5:**
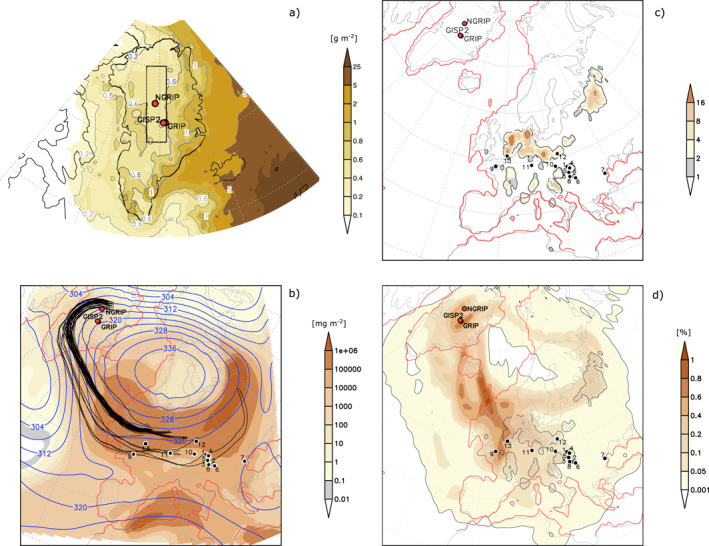
(a) Total simulated dust deposition (g m^−2^) over Greenland accumulated over 30 model years. (b) Trajectories (black lines), total dust load (shading, mg m^−2^) and geopotential height (gpdm) at 700 hPa during an individual dust event in July. (c) Density of emission source points found from backward trajectories based on TOP20 dust deposition events over Greenland when they reach an emission area for the first time (trajectory points per 1°), based on 1,023 trajectory points in total. (d) Trajectory density (percentage of trajectory points per 2° radius) for all TOP20 dust deposition events, based on 24,404 trajectory points in total. Emission areas hatched. Numbering in panels (b–d): 1. Basaharc (Bh, Hungary), 2. Crvenka (Crv, Serbia), 3. Dunaszekcső (Dsz, Hungary), 4. Mende (Me, Hungary), 5. Paks (Pa, Hungary), 6. Titel core site (Tic, Serbia), 7. Urluia (Url, Romania), 8. Zmajevac (Zm, Croatia), 9. La Motte (LMJ, Green Island, Jersey, Channel Islands), 10. Krems‐Wachtberg (KW, Austria), 11. Nussloch (Nus, Germany), 12. Bialy Kosciol (Bk, Poland), 13. Pegwell Bay site (PbE, UK). Red line depicts Last Glacial Maximum land mask based on Paleomodel Intercomparison Project3 guidelines for the 21k experiment.

The 50 most intense dust deposition events (TOP50) over Greenland were selected based on average daily deposition rates over central Greenland (35–45 W; 70–80 N) to analyze the annual cycle of deposition. The TOP20 dust events were additionally analyzed using the LAGRANTO (Sprenger & Wernli, 2015) software package adapted for WRF model output to determine the source areas, transport pathways and transit times from the source areas toward central Greenland. For each of the TOP20 events, a set of 150 backward trajectories, starting at six equidistant levels (200m) up to 1,200 m above ground over central Greenland, were calculated and traced back to the dust source regions. The final set of trajectories per event include only those trajectories passing over a region where actually dust emission occurred at that time. This allows for an estimation of the major transport pathways and transit times of dust particles from Europe toward Greenland over the LGM.

### 2.6 Criteria of Literature Data Selection

New clay mineralogy and Sr–Nd–Hf isotope measurements of PSA samples and NGRIP dust were compared to existing data available in the literature. However, only a small subset of clay mineralogy and isotope data could be used for that purpose. With regard to XRD data, only those measurements were considered that were performed on the clay (<2 μm) fraction of sediments and where compositional data include all clay mineral groups (smectite, illite, chrorite) and kaolinite coming from (semi)quantified spectra. All literature and newly obtained XRD data are normalized to 100. Most of these clay mineral compositions were determined using the method of Biscaye (1965). In some cases, recent sediments are also included in the comparisons (see Dataset S1). Regarding the Sr–Nd–Hf isotope datasets, measurements undertaken on the aluminosilicate fractions of fine dust (<10, <5 or <2 μm) were exclusively considered (see Dataset S2). Isotopic data obtained on other size fractions or acid untreated samples were disregarded. Some of the isotope data are from modern sediments.

## 3 Results

### 3.1 Clay Mineralogy

Clay minerals from the illite group are the most abundant ($\bar{x}$ ± 1SD = 54 ± 6%) phases in LGM dust samples from the GISP2 and GRIP ice core, followed by chlorite ($\bar{x}$ ± 1SD = 27 ± 3%) and kaolinite ($\bar{x}$ ± 1SD = 17 ± 5%), and with minor amounts of smectite ($\bar{x}$ ± 1SD = 2 ± 2%) (Biscaye et al., 1997; Svensson et al., 2000; Dataset S1). Based on XRD data published in the literature and obtained in this study, PSA samples from Alaska, Siberia, Central and SE Asia have similar clay mineral compositions (Figures 2a and 2b; Dataset S1). Other PSAs show more heterogeneous clay mineralogy with samples resembling those of central Greenland ice core dust (some glacial continental North American samples and modern fluvial sediments from Western/Central/East Central Europe and North Africa) and others with smectite‐richer compositions (West Africa, most of North Africa, East Central/Eastern Europe and continental US loess; Figure 2a). A crucial feature is that the GISP2 and GRIP last glacial dust samples have relatively limited variations in terms of clay mineral ratios and plot in the right corner of the chl/kao‐kao/ill‐sme/kao ternary diagram (Figure 2c), resulting from a smectite‐poor composition. Few PSA samples have similar clay mineral ratios, but include Central Asia loess (only those from the literature), SE Asia loess and desert dust (region B), some Alaska and continental US samples and a small number of modern fluvial sediment samples from Western/East Central Europe. A multivariate statistical distance (Text S6 in Supporting Information S1), the so‐called squared Mahalanobis distance (Md^2^) values confirm this picture (Figure S8a in Supporting Information S1) and these sources, most compatible with those of ice core dust, are characterized by Md^2^ < 20–30.

### 3.2 Sr, Nd, Hf, and H Isotope Compositions

The aluminosilicate fractions of LGM dust samples from the NGRIP ice core have narrow ranges in Sr–Nd isotope compositions of 0.719557 – 0.720359 and  −10.74 to  −10.00 *ε*Nd(0). The ^87^Sr/^86^Sr isotope ratios are slightly more radiogenic than those measured for LGM dust from the GISP2 and GRIP ice cores. By contrast, the *ε*Nd(0) values are marginally less radiogenic, but in general the respective values overlap (Figure 3a; Dataset S2). One dust sample of the GISP2 ice core (G2) has outlying Sr–Nd isotope compositions, very likely caused by volcanic contributions. The ^87^Sr/^86^Sr isotope ratios of the leachates of NGRIP dust are lower (0.711551–0.712138) than those of the aluminosilicate fractions, reflecting the less radiogenic Sr isotope composition of sea salt and carbonates removed by acetic acid leaching. The opposite holds true for Nd, for which the removed fraction is relatively more radiogenic (−8.11 to −7.46) than the aluminosilicate one (Dataset S2). Since the isotope composition of the sea salt/carbonate fraction is meaningless for source discrimination of the insoluble mineral dust fraction, these are not considered further in this study. Numerous PSA samples fall in the Nd isotope compositional range of central Greenland ice core aerosols (*ε*Nd(0): about −11 to −9), while a few of them overlap or are close in Sr isotope ratios (Figure 3a). The most compatible PSA samples have Md^2^ values below 8 (Figure S8b in Supporting Information S1) and include samples from SE Asia desert region B and Chinese loess, Northern Mongolia Plateau, Central Asian, continental North American (Nebraska), North African and Eastern/East Central European dust/loess. Some PSAs are much more (Central Asia, SE Asia desert region A, Yukon) or less (West Africa, Alaska, SE Asia desert region C) radiogenic in Nd, others are less (Egypt in North Africa, many SE Asian regions and parts of West Africa) or more radiogenic in Sr isotopic compositions (CLP, Western and Central Europe, parts of North Africa). Volcanic material from Kamchatka (samples KHG and KG) gave unradiogenic ^87^Sr/^86^Sr ratios (about 0.7033) and very radiogenic Nd isotope compositions (*ε*Nd(0): +8–9). Artificial 10:90 mixture of two end‐members (KHG tephra and Luo loess, China) yields Sr–Nd isotope ratios close to ice core dust, while the mixture of 30:70 gave compositions similar to sample G2 of the GISP2 ice core (Figure 3b; Dataset S2).

Hafnium isotope composition of the aluminosilicate fractions of LGM dust samples of the NGRIP ice core range from *ε*Hf(0) of −7.06 to −4.67 (Dataset S2), overlapping with values of two dust samples (132A, B) of the Dye‐3 ice core preindustrial segment (Lupker et al., 2010). These Hf isotope compositions are in agreement with those obtained from some East Central/Eastern European, North African, NE Asian and continental US samples, also indicated by Md^2^ values <10 (Figure S8c in Supporting Information S1). Most of the SE Asian dust samples, including desert region B, Northern Mongolia Plateau and CLP/Beijing loess, are more radiogenic in Hf, with *ε*Hf(0) values varying between −3 and +3 (Figure 3c). The few exceptions include aeolian deposits from Taklimakan and Tengger deserts with *ε*Hf(0) values between −5.17 and −3.71 (samples TD, TD2 and Td25, Dataset S2), which show values at the higher end of ice core dust values. Central Asia loess deposits are clearly more radiogenic in Hf (*ε*Hf(0): −3.03 to −1.99) than NGRIP ice core dust, likewise Yukon loess with *ε*Hf(0) values of +3.82 to +3.96, reflecting volcanic dust additions to these sediments. The KHG and KG pumice fall deposits yield, as expected, highly radiogenic Hf isotope compositions (*ε*Hf(0): +13.97 to +17.03) and the 10:90 and 30:70 mixtures of Chinese loess (Luo) and the KHG sample gave *ε*Hf(0) values of −1.22 and 1.00 (Dataset S2) and both plot far away from NGRIP dust in the Nd–Hf isotope space (Figure 3d).

Hydrogen isotope measurements of clay structural water of two LGM dust samples of the NGRIP ice core yield *δD*_csw_ values of −72.2 and −70.4 ‰ (Dataset S2). Most PSA samples have different, usually more negative *δD*_csw_ values with a range from about −100 to −80 ‰, including Central Asia, SE Asian loess (CLP, around Beijing), Eastern Europe and continental North America (Nebraska loess). The most negative *δD*_csw_ values (−116 to −101) are characteristic for dust samples originating from the northernmost sources (Yukon loess and NE Siberia loess), while the less negative values (−67 to −62 ‰) from North Africa and SE Asian desert region B (Figure 4a). This geographical pattern indicates a latitudinal dependence of *δD*_csw_ in the studied samples and is reminiscent of the modern atmospheric water vapor and rainfall depleted in deuterium toward the poles (Craig, 1961; Dansgaard, 1964). None of the PSA samples match perfectly the NGRIP dust *δD*_csw_ values, but those being close in composition include European loess, SE Asian desert sediments (region B, Taklimakan), CLP loess and North African dust. A small subset of these samples (e.g., Zm from East Central Europe or Td25 from the Taklimakan desert) plot near the NGRIP last glacial aerosols in *ε*Nd(0) versus *δD*_csw_ and *ε*Hf(0) versus *δD*_csw_ spaces (Figures 4b and 4c), and have Md^2^ values of about 16–17 in Nd–Hf–*δD*_csw_ (Dataset S2, Figure S8d in Supporting Information S1).

### 3.3 Dust Transport Simulations

**Figure 6:**
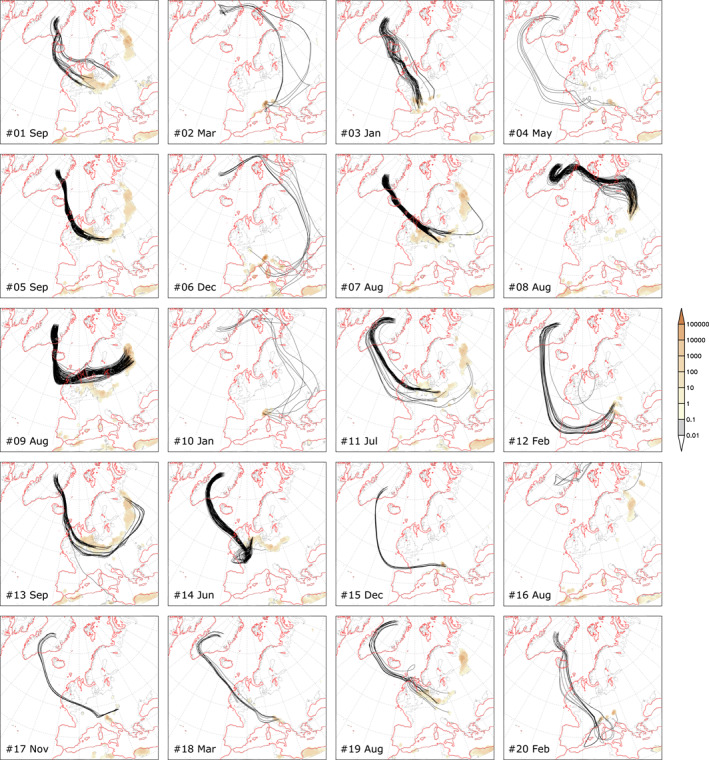
Backward trajectories for the TOP20 deposition events and dust emission areas. Event number and month indicated at the bottom left for each case. Shaded: Mean dust emission [μg m^−2^ s^−1^] between the first and last time a trajectory passed over a region with dust emission for each event.

The 30 year simulations of the LGM dust cycle performed in this study with WRF‐Chem demonstrates that mineral dust emitted from European sources could reach central Greenland during the LGM. Most dust events were found to be triggered by easterly winds, induced by an anticyclonic circulation around a semi‐permanent high‐pressure system over the Fennoscandinavian ice sheet (Ludwig et al., 2016). Dust particles were then transported toward the west and became involved in the cyclonic circulation of low‐pressure systems over the North Atlantic, which finally directed dust toward Greenland, where deposition took place (Figures 5a–5c). Another dust transport route to the east of the Fennoscandinavian high‐pressure center is also identified in the model simulations (Figures 5d and 6 and Figure S7 in Supporting Information S1), albeit with subordinate occurrence. Interestingly, trajectories reached back to North Africa for one of the 20 most intense (TOP20) dust events (Figure 6). Major source regions of the TOP20 dust events include the Po Plain and the NW Adriatic Sea region, the Carpathian Basin, the northern part of the East European Plain, and intense dust emission hotspots from the North European Plain to the North Sea continental shelf (Figures 5c and 6). The TOP20/TOP50 dust events have two seasonal maxima during winter/early spring and late summer/early autumn (Table S3 in Supporting Information S1). Mean transit time calculated from model outputs is 4.68 days for the TOP20 dust events, with the shortest and longest transit times of 2.0 and 7.75 days (Table S4 in Supporting Information S1). The total simulated dust deposition over central Greenland for the 30 model years is ∼0.53 g m^−2^ (Figure 5a), translating to a dust deposition flux of 17.7 mg m^−2^ yr^−1^.

## 4 Discussion

Source discrimination of aerosols emitted from potentially multiple continental areas, entrained at various heights in the atmosphere and subsequently deposited on polar ice sheets in the past is inherently complex. The reliability of provenance interpretations largely depends on the dust source tracers applied, the general understanding of the major controls on these indicators, and the analytical uncertainties and reproducibility of tracer data. We first discuss these issues prior to considering the interpretation of these data in terms of dust source.

### 4.1 Uncertainties Related to Dust Tracers

In this study, one of these dust fingerprints used in source identification is the clay mineralogy of PSAs and ice core dust. The distribution of clay minerals on the continents is controlled by the local lithology, drainage characteristics, weathering and climate, and it is latitude dependent (Biscaye, 1965; Biscaye et al., 1997; Griffin et al., 1968). Therefore, each dust source has a characteristic spectrum of clay minerals and the abundances and abundance ratios may be source diagnostic. It is of note that the XRD intensity‐based calculation of the composition of a given sample in weight percent of mineral phases is semi‐quantitative, with results being affected by ±10% uncertainties for major and ±20% for minor constituents (<20 wt.% amount) and with detection limits of about 1 wt.%–5 wt.% (Moore & Reynolds, 1997).

Other traditional dust provenance indicators include the ^87^Sr/^86^Sr and ^143^Nd/^144^Nd isotope ratios of the carbonate‐free, aluminosilicate fractions of dust (Grousset & Biscaye, 2005). The Sr isotope composition of aeolian dust is controlled by carbonates, feldspars and various clay minerals (Brass, 1975; Capo et al., 1998) and there is a strong coupling between Sr isotopic ratios and grain size in sediments (Chen et al., 2007; Dasch, 1969; Feng et al., 2009; Meyer et al., 2011). In ice core dust source discrimination using Sr isotopes, calcite and gypsum aerosols being entirely of marine origin are removed by acid treatment (Biscaye et al., 1997; Svensson et al., 2000), as these phases are capable of masking the Sr isotope composition of the aluminosilicate (or lithic) fraction that is of interest here (Delmonte et al., 2004). Furthermore, the <10 or <5 μm particle size fractions representative of the grain size of ice core dust are compared (Figure S2 in Supporting Information S1; Biscaye et al., 1997; Bory, Biscaye, Piotrowski, & Steffensen, 2003; Svensson et al., 2000), thereby minimizing grain size effects. However, the use of different acids (hydrochloric or acetic acid) in various concentrations and different reaction times may strongly influence the measured Sr isotope ratio due to the partial (calcite) or complete (calcite/dolomite) removal of carbonates and even a fraction of clay minerals (Asahara et al., 1999; Yokoo et al., 2004). Re‐measurements of new separations of loess samples previously prepared and measured for Sr isotope ratios by Újvári et al. (2015) demonstrate that subtle deviations in grain size (Figures S2a in Supporting Information S1) may result in significant differences in ^87^Sr/^86^Sr isotope ratios (0.0032–0.0059; Figure S3a in Supporting Information S1). This implies that extreme care must be exercised when the Sr isotope compositions published in different studies are to be compared and it is important to strictly follow the same methodology (acid treatment, grain size fraction) in all these studies to reach meaningful interpretations using Sr isotopes. By contrast, ^143^Nd/^144^Nd isotope compositions, controlled by allanite, apatite, garnet, monazite, sphene, zircon and also plagioclase, amphibole, biotite and clay minerals (Aubert et al., 2001; Viers & Wasserburg, 2004), are much less or not at all affected by these issues (0.000010–0.000026 or 0.2–0.5 *ε*Nd units, Figure S3b in Supporting Information S1). These 0.2–0.5 *ε*Nd unit variations are comparable to the analytical reproducibility (about 0.3 *ε*Nd units) of Nd isotope measurements as determined by replicate analyses of three aeolian sediment samples (Figure S4 in Supporting Information S1).

Hafnium isotope ratios have only been used once, to our knowledge, in ice core dust source discrimination on the Dye‐3 ice core (Lupker et al., 2010), partly because of analytical issues, low amount of ice core dust available and the scarcity of ^176^Hf/^177^Hf data from the fine fractions of PSA samples. Bulk samples of wind‐blown sediments are dominated by zircons having extremely high Hf contents (Hoskin & Schaltegger, 2003) and unradiogenic ^176^Hf/^177^Hf ratios (Újvári & Klötzli, 2015), while the clay fractions contain little zircon (Újvári et al., 2018). This is further evidenced in Figure S16 of Supporting Information S1 (Text S8 in Supporting Information S1), where zircon depletion toward the finer grain size fractions of Quaternary aeolian loess, paleosol and red clay samples is clearly visible in the Zr, Hf concentration and Nd–Hf isotope data sets. The more radiogenic Hf isotopic compositions of the clay fraction (Zhao et al., 2015) are controlled by clay minerals that incorporate radiogenic Hf released from higher Lu/Hf phases (apatite, garnet, xenotime and sphene) during incongruent silicate weathering (Bayon et al., 2009; van de Flierdt et al., 2007). In this study, the ammonium‐bifluoride method was used, which allowed for a fast and complete dissolution of a large number of 5–10 mg samples for Hf–Sr–Nd isotope analyses (Újvári et al., 2021), including NGRIP ice core dust. Reproducibility of Hf isotope analyses was found to be between 0.3 and 0.8 *ε*Hf units, translating to differences of about 0.000009–0.000023 in ^176^Hf/^177^Hf isotopic ratios based on replicate measurements of three aeolian dust samples (Figure S4 in Supporting Information S1). These are usually within or slightly higher than analytical errors of individual measurements.

The *δD*_csw_ values of the <5 and < 2 μm fractions of ice core dust and PSA samples are thought to be controlled by the D/H isotope compositions of various hydrous silicates in the clay‐mineral size fraction of these samples. The D/H isotope composition of hydrous minerals depends on the isotopic composition of ambient water and the temperature at the time of mineral formation, as well as the mineral‐specific isotopic fractionation factor at the temperature of formation (under isotopic equilibrium), and finally whether the mineral has retained its original isotopic composition (Savin & Hsieh, 1998). In the case of clay minerals formed by surficial weathering, the ambient water would be meteoric water and temperatures would approximate Earth's surface temperatures. The dust samples studied are thought to be primarily made up of detrital clay minerals, largely by clays formed in‐situ by chemical weathering that may have a range of formation ages and origin. This suite of clay minerals with diverse D/H isotope compositions defines *δD*_csw_ values characteristic for the different PSAs. Indeed, there is a spatial pattern in *δD*_csw_ of PSA samples, with the most negative values for dust samples from higher latitudes (Figures 4a, Dataset S2), and some PSAs have a set of *δD*_csw_ values sufficiently well‐defined to be diagnostic of their source. For the source discrimination to be meaningful, the reproducibility of D/H isotope measurements must be considered. This was found to be about 1–4 ‰ within the <5 and < 2 μm fractions and mostly range between 3 and 9 ‰ when these two fractions are compared (Figure S6 in Supporting Information S1). Thus, sources with *δD*_csw_ values in the range of −71.3 ± 9 ‰ (mean ± reproducibility of NGRIP dust, Dataset S2) must be considered as potential contributors to last glacial dust of central Greenland.

### 4.2 Last Glacial Greenland Dust Sources

#### 4.2.1 Low(er) Probability Single Source Regions and Source Combinations

Based on the suite of dust tracers applied here and considerations outlined above, some PSAs have a low probability of being the source of last glacial dust to central Greenland. While Alaskan loess samples are among the most compatible with central Greenland last glacial dust in terms of clay mineralogy, direct sourcing (i.e., without mixing) of dust from Alaska has been excluded by Biscaye et al. (1997) based on Sr–Nd isotopic compositions. The Sr–Nd isotope ratios here indeed support the exclusion of the Alaskan source (Dataset S2), although more Alaskan sediments would need to be analyzed to further confirm this. The Yukon loess, as a single source, is likewise incompatible with central Greenland dust, as it has much less radiogenic Sr and more radiogenic Nd isotope compositions (Zdanowicz et al., 2006), positive Hf isotope ratios and very negative *δD*_csw_ values compared to Greenland dust (Dataset S2, Figures 3a and 3c and 4). The Sr–Nd–Hf isotopic compositions of Nebraska loess fine fractions are similar to central Greenland last glacial dust (Újvári et al., 2015; Dataset S2), but these sediments are usually extremely smectite‐rich and poor in chlorite and kaolinite (Újvári et al., 2015, Dataset S1). Smectite aggregation and resulting fractionation during atmospheric transport (Scheuvens et al., 2013; Singer et al., 2004) would not lead to the almost total loss of such high amounts of smectites (about 70–75 wt%) and chlorite and kaolinite enrichment during atmospheric transport can likewise be excluded, unless mixed with another chlorite and kaolinite rich dust source. Although direct derivation of last glacial ice core dust from this PSA is thus unlikely, sourcing of ice core dust from the US drylands (e.g., Nevada; Aarons et al., 2017) based on Nd–Hf isotopic compositions cannot entirely be ruled out, but this requires a more systematic study of the fine, lithic fractions of last glacial dust deposits of that region.

Although direct dust transport to Greenland from single North American sources is unlikely based on the data here, the possibility of a contribution as part of a mixture with dust from other sources must also be considered, especially because the atmosphere over Greenland was shown to be well‐mixed during colder periods of the last glacial cycle (Mayewski et al., 1994). Indeed, given the previously modeled SE Asian dust transport pathway to Greenland (Andersen et al., 1998; Mahowald et al., 2011), North American glacially sourced aerosols could potentially have mixed with SE Asian dust en route to Greenland. However, given the very negative *δD*_csw_ values of these sources (SE Asia: mostly <–75 and North America: <–90 ‰) and the NGRIP dust average (−71.3 ‰), this possibility is quite unlikely. In terms of *δD*_csw_ values, a low latitude source with higher than ∼–70.0 ‰ such as North Africa would be needed to reproduce NGRIP ice core dust compositions. However, North African transport routes were northward during the glacial, as shown in our modeling results detailed later (see Figure 6), meaning that mixing of SE Asian‐North American and North African dust in a single pathway is implausible. Despite this, our Monte Carlo (MC) simulations reveal that the Sr–Nd–Hf isotopic compositions of central Greenland last glacial dust is relatively well explained by mixing North African dust with Nebraska loess (Figure S9a and S9b in Supporting Information S1), in contrast to the Yukon loess, although the *δD*_csw_ values are more difficult to reproduce (Figure S9c and S9d in Supporting Information S1). However, it is important to emphasize that all four isotopic compositions must be considered together in assessing plausible mixing ratios for dust admixtures. Indeed, mixing ratios that simultaneously reproduce all the isotopic compositions (Sr–Nd–Hf and *δD*_csw_) of ice core dust well are very few, with 10 and 2 such combinations found out of 1,000 modeled cases for the Nebraska and Yukon loess, respectively (Table S7 in Supporting Information S1). Of those few mixing ratios, none yield modeled clay mineral compositions compatible with last glacial ice core dust; all show much more smectite, less illite and kaolinite and much less chlorite compared to ice core dust (Figure S14 in Supporting Information S1). For these reasons, and based on the currently available data, we believe that the likelihood of North American sources contributing significantly to the last glacial ice core dust deposited in central Greenland, even when mixed with other sources, is low.

Central Asian dust sources, represented by loess from Tajikistan, Kazakhstan and western China (Ili Basin), are also considered unlikely as direct single contributors of aerosols to central Greenland over the LGM due to their more radiogenic Nd–Hf isotope compositions and low *δD*_csw_ values (Figures 3a and 3c and 4, and Figure S8d in Supporting Information S1). The NE Asian (Siberia) region is underrepresented by only one loess sample, which has clay mineralogy and Nd–Hf isotope compositions (Figures 2 and 3 and Figure S8a, S8b) resembling central Greenland ice core dust. However, its more radiogenic ^87^Sr/^86^Sr isotope ratio and very negative *δD*_csw_ values (down to −115 ‰; Figure 4) suggest that NGRIP last glacial dust was unlikely to be directly derived from this Siberian source.

These indications of North American/Siberian sources being at least partly incompatible with central Greenland last glacial aerosol compositions imply that despite being important in simulations, certain modeled LGM dust sources (Lambert et al., 2015; Mahowald et al., 2006; Werner et al., 2002) do not seem to contribute dust to central Greenland. A possible explanation for the incompatibility with North American sources, despite the large interior continental planes and upwind location to Greenland, could be that the North Atlantic jet stream and storm tracks were more zonally oriented and less variable at the LGM compared to today (Löfverström, 2020; Löfverström et al., 2016).

#### 4.2.2 High(er) Probability Single Source Regions and Source Combinations

Derivation of last glacial ice core dust from North Africa was excluded by early studies (Biscaye et al., 1997; Svensson et al., 2000), but later considered plausible (Han et al., 2018; Meyer et al., 2017). In general, western/central Saharan dust samples have abundant kaolinite and smectite (Dataset S1, Figure 2; Scheuvens et al., 2013), while some recent fluvial sediment samples around the High Atlas in Morocco have smectite‐poor compositions similar to LGM ice core dust. There is a wide variety in isotopic compositions of North African/Saharan dust samples with ^87^Sr/^86^Sr of 0.730 to 0.708, εNd(0) of −18.5 to −4 and εHf(0) values of −13.7 to +3.5, controlled by lithologies of the West African craton (Mali) to young volcanic rocks in Egypt (Abouchami et al., 2013; Grousset & Biscaye, 2005; Zhao et al., 2018). Our isotopic data, together with those presented by Zhao et al. (2018), do not exclude the possibility of sourcing of, or at least an admixture to, ice core dust from the northernmost part of Africa (Morocco/Tunisia) (e.g., sample Mao, Datasets [Link], [Link]). This is especially intriguing in the light of our model simulation results, which include some backward trajectories over Europe reaching back to North Africa (Tunisia, Figure 6, Figure S7 in Supporting Information S1).

Out of the analyzed PSAs, some specific regions of SE Asia and Europe are the most plausible single sources of last glacial dust of central Greenland ice cores. Clay mineralogical compositions of most SE Asian loess/desert sediment samples match relatively well those of last glacial dust from the GISP2/GRIP ice cores (Figure 2, Table S5 in Supporting Information S1), usually with higher illite content of up to 77% (Taklimakan, Tengger desert) and less abundant chlorite (Dataset S1). However, few SE Asian sources are compatible with last glacial ice core dust in terms of isotope compositions. The SE Asian desert region A (northern deserts, including the Gobi) and the Northern Mongolia Plateau samples are usually less radiogenic in Sr isotope and more radiogenic in Nd isotope composition, and loess deposits from the CLP and around Beijing have higher ^87^Sr/^86^Sr (Figure 3a). This latter discrepancy was explained by volcanic dust additions to SE Asian aerosols and mixing in 10/90% proportions (Biscaye et al., 1997), which is tenable based on the Sr–Nd isotope data (Figure 3b). However, some dust samples from the Taklimakan and Tengger desert (desert region B) have overlapping Sr–Nd isotope compositions with GISP2/GRIP (partly by NGRIP) dust, and thus, volcanic dust admixture is not required to explain ice core dust ^87^Sr/^86^Sr and ^143^Nd/^144^Nd ratios.

Hafnium isotope compositions put further constraints on plausible sources, as most PSAs of SE Asia have much more radiogenic ^176^Hf/^177^Hf isotope ratios (εHf(0): −3 to +4) than NGRIP ice core dust (εHf(0): −7.06 to −4.67). Few exceptions include Tengger (TD/TD2) and Taklimakan (Td25) desert samples with εHf(0) values of −5.17 to −3.71, being at the higher end of NGRIP ice core dust compositions (Figure 3c). Importantly, the Hf isotope ratios also demonstrate that the previously proposed mixing model of circum‐Pacific volcanics and SE Asian dust is untenable. This confirms what has already been proposed by Svensson et al. (2000) that an almost constant (10%) contribution of volcanic material, inferred from the restricted range of Sr isotope ratios of central Greenland dust, seems unlikely considering the stochastic nature of volcanic eruptions. Measurements of Kamchatka volcanics (KHG and KO tephra) and artificial mixtures of the KHG tephra, which has been identified in Holocene ice of the NGRIP core (Cook et al., 2018), and loess from the Luochuan loess‐paleosol sequence (Luo) indicate that an admixture of volcanic material to SE Asian dust would further increase the already radiogenic ^176^Hf/^177^Hf isotope ratios of SE Asian dust, shifting the composition of the mixtures in the opposite direction compared to those of the NGRIP last glacial dust (Figure 3d). Thus, this mixing model can safely be excluded as an explanation for ice core dust composition.

By contrast, direct sourcing of last glacial dust of central Greenland from the Taklimakan/Tengger deserts is supported by the D/H isotope compositions. The *δD*_csw_ values of NGRIP dust are different from most SE Asian dust samples (Figure 4), but within reproducibility overlap with one sample from the Taklimakan (Td25) and another from the CLP (Dataset S2). Additional support for SE Asian deserts being the major contributors of dust to the Greenland Ice Sheet over the LGM comes from the fact that they are upwind to Greenland and their high altitude, facilitating uplift of dust into the long‐range transporting, westerly wind regime (Svensson et al., 2000). Further evidence is the origin of volcanic material in Greenland ice. Tephra analyses of Greenland ice cores indicate that beyond the clear dominance of relatively proximal Jan Mayen and Icelandic sources, volcanic ash from the Pacific Arc was repeatedly entrained to and deposited in Greenland, whereas no ash particles from central Europe have been identified in Greenland ice cores despite that region being very active in volcanism during the last glacial period (Abbott & Davies, 2012; Bourne et al., 2016).

An alternative hypothesis for the origin of last glacial dust in Greenland is the derivation from European sources (Újvári et al., 2015), which has recently been dismissed by Han et al. (2018) based on Sr and Pb isotopic considerations. Unfortunately, the rejection of this hypothesis was based on bulk sediment Sr–Pb isotopic data from Europe (Han et al., 2018) disregarding the fact that both Sr (Chen et al., 2007; Dasch, 1969; Feng et al., 2009; Meyer et al., 2011) and Pb isotope compositions (Feng et al., 2010) are grain size‐dependent. The large variability in Sr–Pb isotope compositions between the bulk and clay fractions (e.g., Δ^206^Pb/^207^Pb and Δ^208^Pb/^207^Pb: 0.01–0.04; Figure S17 in Supporting Information S1) reaches and may even exceed differences in isotopic compositions between ice core dust and European loess, thereby introducing a significant uncertainty and preventing meaningful source identification. As such, the data presented in Han et al. (2018) cannot be used in testing European dust sources to Greenland. Our new data further suggest that the hypothesis of European dust transport to Greenland cannot be ignored. The clay mineralogy of most glacial loess samples from Europe are relatively smectite‐rich (Figure 2). This is especially true for East Central/Eastern European loess dominated by local sources of the Carpathians/Carpathian Basin (Fu et al., 2021), also seen in modern river sediments of the Danube catchment (Martinez‐Lamas et al., 2020). However, tributaries of the Danube River draining the Alps show smectite‐poor compositions close to those of ice core dust (Figure 2, Figure S8a in Supporting Information S1) and some loess deposits, for instance along or near the Drava River, also have lower smectite contents of about 20%–25% (e.g., sample Zm, Dataset S1). Considering the uncertainties of semi‐quantitative XRD data and that smectite fractionation during transport cannot be ruled out (see discussion above), derivation from East Central Europe is still a possibility. This is even more so because Taklimakan dust has about 20% higher illite content compared to last glacial dust from central Greenland ice cores, so the direct sourcing of ice core dust from the Taklimakan would likewise require illite loss during atmospheric transport. Another option, assuming that the particulate matter retains its original mineralogical composition during atmospheric transport, is that the dust mixed with other sources to produce the final composition of the dust in the ice cores. This situation clearly demonstrates the ambiguities related to the use of mineral tracers alone, which in many cases is misleading (Fu et al., 2021) and does not allow for robust source discrimination. The multi‐tracer approach of this study reveals this complexity and highlights the importance of using isotope tracers as well. In Sr–Nd isotopic space, some East Central and Eastern European samples plot close to central Greenland ice core dust (Figure 3a, S8b in Supporting Information S1), while the majority of samples are more radiogenic in ^87^Sr/^86^Sr. Part of the East Central/Eastern European loess samples overlap with the NGRIP dust samples in Hf isotopic compositions, while those from Western (ice marginal) Europe are usually less radiogenic compared to ice core dust (Figure 3c, Table S5 in Supporting Information S1). In terms of D/H isotope compositions, glacial dust from Western and East Central Europe overlap the range measured for NGRIP last glacial dust (Figure 4), and some European glacial loess samples (especially sample Zm, Datasets [Link], [Link]) are very similar to NGRIP last glacial aerosols in Sr–Nd–Hf and D/H isotope compositions (Figure S8c and S8d in Supporting Information S1).

Beyond this tracer evidence for potential European dust contributions, recent studies demonstrate higher dust mass accumulation rates in Europe than in China over the LGM (Rousseau et al., 2021) and that the variations in glacial dust deposition on centennial–millennial timescales in Europe and Greenland were synchronous within uncertainty on radiocarbon chronologies (Moine et al., 2017; Újvári et al., 2017). More southerly positions of the upper‐tropospheric, eddy‐driven Atlantic jet during cold, dusty stadials (Löfverström et al., 2016; Újvári et al., 2017) in combination with anticyclonic circulation over the Fennoscandian Ice Sheet induced by a semi‐permanent high‐pressure system (Ludwig et al., 2016), would have promoted dust transport from European sources to Greenland. At the same time, dust flux estimates based on compiled optically stimulated luminescence ages from multiple Chinese and Central Asian loess sites suggest high dust accumulation during the late LGM (23–19 ka) and much lower for the early LGM (26.5–23 ka) in these areas (Cheng et al., 2021; Kang et al., 2015), in contrast to what has been measured in Greenland ice cores (Rasmussen et al., 2014). However, a reduction or absence of dust accumulation in an area does not necessarily imply lower dust production and emission by the same area. Indeed, previous research argues that the CLP is a highly dynamic environment which may have resulted in substantial internal aeolian recycling and erosion of pre‐deposited material (Kapp et al., 2015; Licht et al., 2016; Stevens et al., 2018).

Results from our high spatial resolution regional model simulation further reduce the ambiguities about the possibility of dust transport from Europe to Greenland over the LGM, and clearly demonstrate that aerosols emitted from several European glacial dust hotspots could reach the Greenland Ice Sheet in all seasons, albeit with varying seasonal occurrence (Table S3 in Supporting Information S1, Figure 6), resulting in a dust deposition flux of 17.7 mg m^−2^ yr^−1^ in central Greenland. This is, however, much lower than the GS‐2.1 stadial dust flux for the 21–22 ka period (∼102–203 mg m^−2^ yr^−1^, e.g., Serno et al., 2015), for which the model was run, and the GS‐3 stadial peak dust deposition at 25.8 ka (498 mg m^−2^ yr^−1^), while it is comparable with the GI‐3 and 2.2 interstadial values of ∼20 mg m^−2^ yr^−1^. The lower simulated dust flux values may be due to the fact that the strength of European dust sources and dust emission values in the model have not been calibrated with actual dust accumulation values, for example, using observed loess sediment accumulation rates. However, this does not affect the main dust transport pathways or their characteristics such as directions and seasonal distributions. The modeled transit times of 2.0 and 7.75 days (mean of 4.68 days) of the TOP20 events are obviously much shorter than those considered in earlier studies for the LGM transport of calcium (Ca^2+^) from East Asian sources (Schüpbach et al., 2018), and would have important implications for interpreting the variability of the Ca^2+^ record of ice cores in terms of significance of drivers either related to sources (strength, storminess, aridity) or atmospheric transport (residence time, wet/dry deposition, transport distance) (Werner et al., 2002). Modeled trajectories crossing Europe and reaching back to North Africa imply that mixing of dust from these sources may have been possible, further complicating the already complex problem of ice core dust provenance Indeed, our MC simulations with two component mixtures of North African and European dust demonstrate that both the isotopic compositions (Figure S10, Table S8 in Supporting Information S1) and clay mineralogy (Figure S15 in Supporting Information S1) of last glacial dust from central Greenland ice cores can be reproduced. Further simulations indicate that the isotopic compositions of last glacial ice core dust can be explained relatively well by the mixing of dust from SE Asian desert regions and the CLP (Figure S11 in Supporting Information S1) or even three distinct PSAs of the NH, including the Taklimakan, European, and North African sources (Figures S12–S13 in Supporting Information S1). In the present study, the PSAs considered most likely by previous geochemical and modeling studies have been investigated, but we cannot exclude that others may have existed. Also, the possibility that aerosols from more than two or three distinct sources may have been mixed also cannot be excluded, but modeling of such mixtures has not been undertaken in the present study as this would require additional dust tracer datasets from even more PSAs of the NH.

## 5 Conclusions and Outlook

We present here the most in‐depth and multi‐proxy analysis of last glacial dust sources to Greenland to date, supported by a regional model simulation of the dust cycle, including the computation of dust trajectories. Our datasets reveal that previous claims of well‐constrained, specific Greenland dust sources are not supported. Rather, the data reveal three plausible, competing hypotheses for the origin of LGM dust recovered from central Greenland ice cores. Provided that ice core dust is from single sources, still the most likely scenario is that these aerosols are directly derived from the Taklimakan and/or Tengger desert, while other SE Asian sources are excluded. However, based on the compositional and modeling data presented, sourcing of ice core dust from Europe is also plausible, and furthermore, an admixture with North African sources cannot be ruled out. To gain more insight into Greenland ice core dust provenance, further, wide‐scale investigation of many more PSA samples would be required, together with high spatial resolution dust cycle simulations for the entire NH over the LGM. These models should preferably be calibrated with observed dust accumulation rates, for example, using absolutely dated loess deposits on the continents (Kohfeld & Harrison, 2001; Mahowald et al., 2006; Stevens et al., 2016; Újvári et al., 2010, 2017), to accurately simulate terrestrial dust source strengths and deposition flux in central Greenland. In addition, further isotope geochemical studies of other sections of the central Greenland ice cores with lower dust accumulation would be worthwhile to better understand dust‐climate feedbacks. In part, such studies have already been carried out (Han et al., 2018; Svensson et al., 2000) and found low variability in dust Sr–Nd–Pb isotopic compositions over time, suggesting that if Greenland had had multiple dust sources, their relative contributions have been fairly well preserved during the abrupt climate transitions of the last glaciation. However, additional information from as many isotopic dust tracers as possible is still required from specific periods of the last glaciation. As such, targeting intervals of high NH summer insolation, when a low Sahara‐Sahel signal is expected (“Green Sahara” phases/humid North Africa; e.g., Tjallingii et al., 2008) and targeting lows in NH summer insolation when the opposite would be expected, could be a way of better quantifying the potential contributions from north African sources and deconvolving the dust provenance signal in the central Greenland ice core records.

## Supporting information

Supporting Information S1

Data Set S1

Data Set S2

Data Set S3

Data Set S4

## Data Availability

The data that supports the findings of this study are published in the main text and Supporting Information S1 of this article and are openly available in the PANGAEA Data Repository (Újvári, 2021).
