# Supplementary material for: Greenland Ice Core Record of Last Glacial Dust Sources and Atmospheric Circulation
Source: J Geophys Res Atmos. 2022 Aug 5;127(15):e2022JD036597. doi: 10.1029/2022JD036597 (PMC9542552; doi:10.1029/2022JD036597)
Supplement: Supplementary file 1 — Supporting Information S1 [file JGRD-127-e2022JD036597-s005.docx]

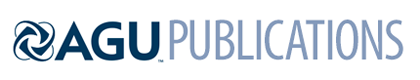


*Journal of Geophysical Research: Atmospheres*

Supporting Information for

**Greenland ice core record of last glacial dust sources and atmospheric circulation**

G. Újvári^1,2,3*^, U. Klötzli^3^, T. Stevens^4^, A. Svensson^5^, P. Ludwig^6^, T. Vennemann^7^, S. Gier^8^, M. Horschinegg^3^, L. Palcsu^9^, D. Hippler^10^, J. Kovács^11,12^, C. Di Biagio^13^, P. Formenti^13^

^1^Institute for Geological and Geochemical Research, Centre for Astronomy and Earth Sciences, Eötvös Loránd Research Network, Budaörsi út 45,1112 Budapest, Hungary

^2^CSFK, MTA Centre of Excellence, Konkoly Thege Miklós út 15-17, 1121 Budapest, Hungary

^3^Department of Lithospheric Research, University of Vienna, Althanstrasse 14, 1090 Vienna, Austria

^4^Department of Earth Sciences, Uppsala University, Villavägen 16, 75236 Uppsala, Sweden

^5^Physics of Ice, Climate and Earth; Niels Bohr Institute; University of Copenhagen, Tagensvej 16, 2200 Copenhagen, Denmark

^6^Institute for Meteorology and Climate Research, Karlsruhe Institute of Technology, Wolfgang-Gaede-Strasse 1, 76131 Karlsruhe, Germany

^7^Institute of Earth Surface Dynamics, University of Lausanne, Géopolis 4634, 1015 Lausanne, Switzerland

^8^Department of Geology, University of Vienna, Althanstrasse 14, 1090 Vienna, Austria

^9^Isotope Climatology and Environmental Research Centre, Institute for Nuclear Research, Bem square 18/c, 4026 Debrecen, Hungary

^10^Institute of Applied Geosciences, Graz University of Technology, Rechbauerstrasse 12, 8010 Graz, Austria

^11^Environmental Analytical and Geoanalytical Research Group, Szentágothai Research Centre, University of Pécs, Ifjúság u. 20., 7624 Pécs, Hungary

^12^Institute of Geography and Earth Sciences, University of Pécs, Ifjúság u. 6., 7624 Pécs, Hungary

^13^Université de Paris and Univ Paris Est Creteil, CNRS, LISA, F−75013 Paris, France

*Corresponding author: G. Újvári (email: [ujvari.gabor@csfk.org](mailto:ujvari.gabor@csfk.org))

**Contents of this file**

Supplementary text S1 to S9

Figures S1 to S17

Tables S1 to S9

**Additional Supporting Information (Files uploaded separately)**

Supporting Datasets S1, S2, S3 and S4 (Excel files)

**Introduction**

This file contains data and descriptions of the exact positions of NGRIP last glacial dust samples, particle size separation methods, reproducibility of isotopic measurements (Sr-Nd-Hf, δD), dissimilarity measure and mixture calculations, provides additional information to dust transport simulations and the evaluation of Pb isotope variations in aeolian dust samples.

**Text S1. NGRIP ice core samples**

Four samples of the NGRIP ice core from a depth interval of 1829.85-1818.30 m were analyzed in this study (Figure S1), representing ~32-38 yrs of accumulation during the dustiest period of the Last Glacial Maximum (LGM). Measured weights of bulk dust in bags 3306-3307, 3323, 3326 and 3327 were 17.30, 13.53, 10.17 and 16.01 mg. Treatment of samples using 0.5 mol/L acetic acid (1 hr of reaction) removed 7.73, 6.44, 4.64 and 7.46 mg of soluble salts and carbonates, called leachates (L) in Supporting Dataset S2. The remaining masses of the aluminosilicate fractions were measured to be 9.57, 7.09, 5.53 and 8.55 mg in bags 3306-3307, 3323, 3326 and 3327.


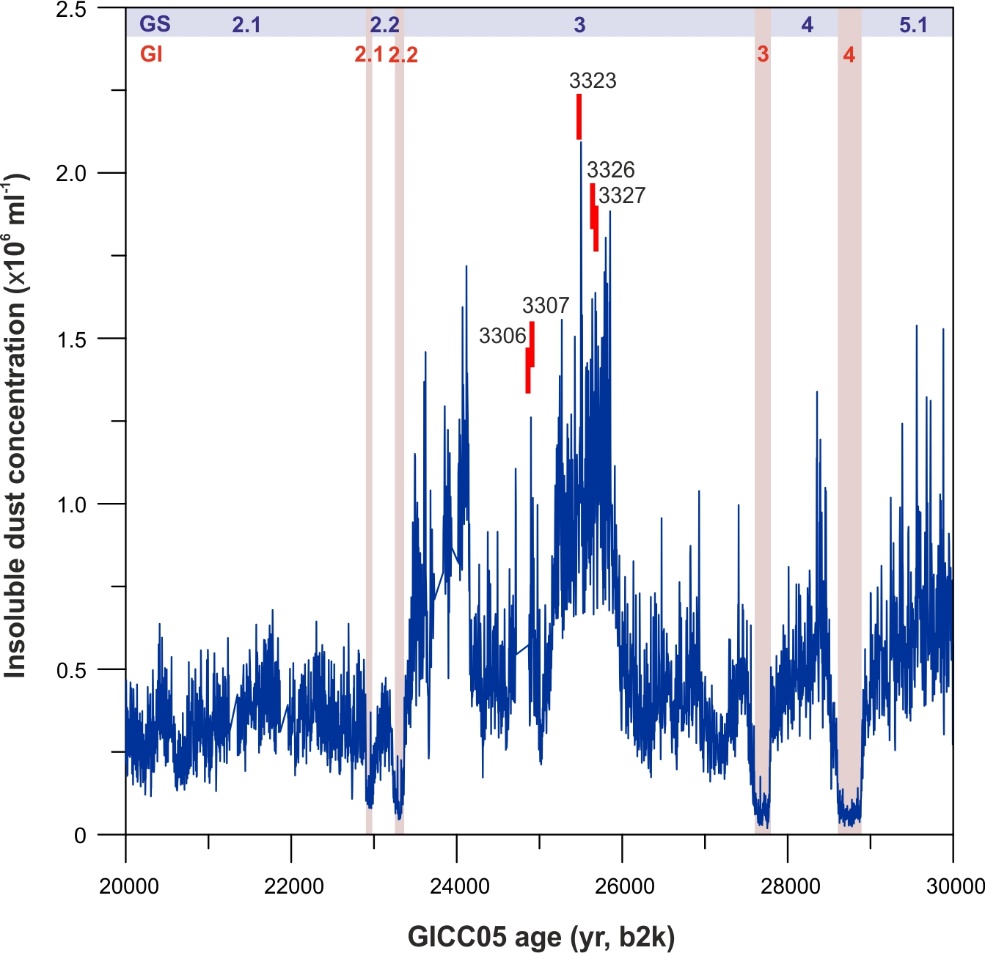


**Figure S1**. NGRIP ice core dust sample positions on the GICC05 age scale and the insoluble dust concentration record (number of particles >1 μm*10^6^ ml^-1^). Greenland stadial and interstadials (GS/GI) after Rasmussen et al. (2014).

**Text S2. Particle size separation of potential source area (PSA) samples**

The collected PSA samples were size separated using hydrophobic <5 µm Mitex filters or by wet sedimentation to properly isolate the <5 and <2 µm fractions for isotopic analyses. The settling method is based on Stokes’ Law and the withdrawal times and depths were 3 and 24 hrs and 10 and 5 cm for the separation of the <5 and <2 µm fractions, respectively. As demonstrated in Figure S2, the particle size distributions (PSDs) of the PSA sample size separates (panel a) closely match that of LGM dust from the NGRIP ice core (panel b). Separations done by Újvári et al. (2015) are also shown for comparison.


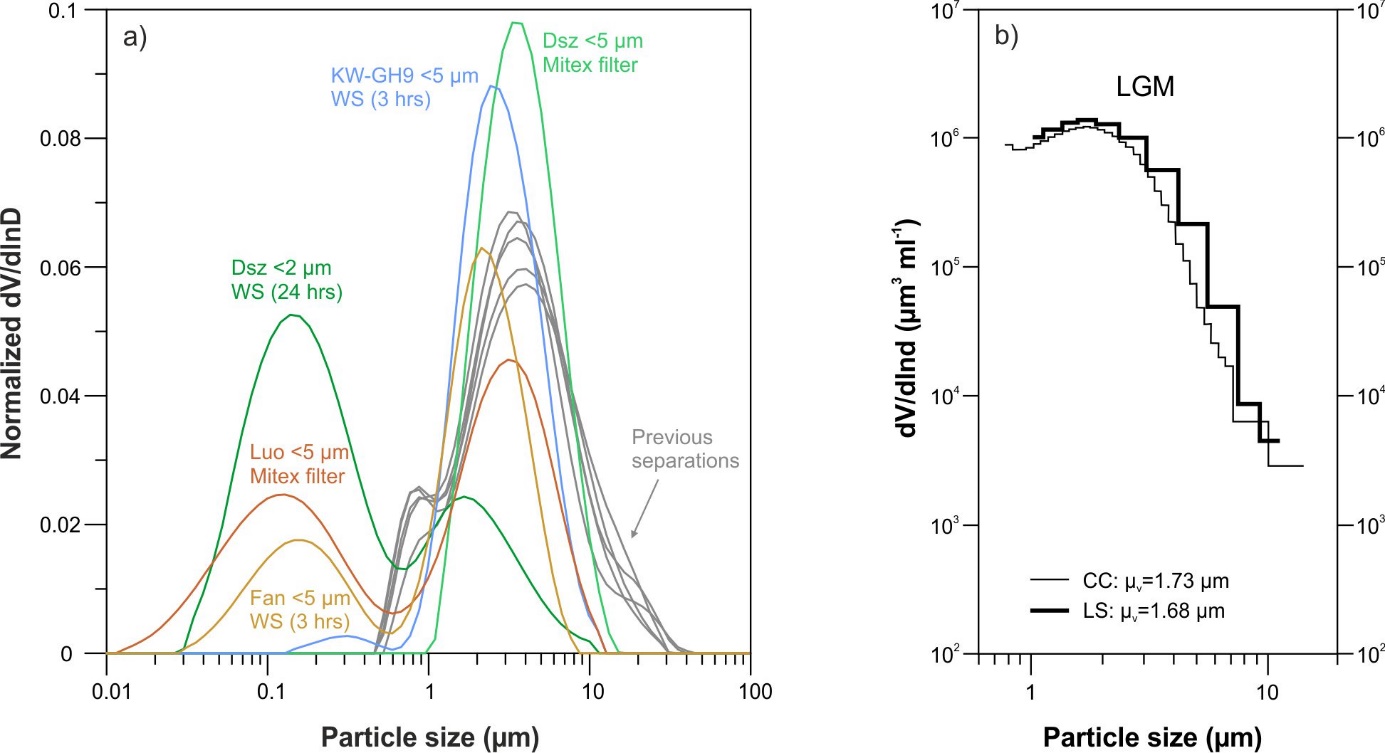


**Figure S2.** Particle size distributions (PSDs) of A) various separates of PSA samples and aerosols recovered from the LGM section of the NGRIP ice core. Size separations of PSAs were undertaken by wet sedimentation (WS, duration defined in hours) and using hydrophobic Mitex filters. The aerosol size data of the NGRIP ice core are from Ruth et al. (2003). PSDs were determined by laser diffraction (Malvern Mastersizer 2000 and 3000 instruments) for PSAs (panel a) and by laser light attenuation (laser particle sensor, LS) and Coulter Counter (CC) for NGRIP dust (panel b). The mode (μ_v_) of PSDs of NGRIP dust is shown for laser sensor and Coulter Counter measurements. PSA samples are loess from Dunaszekcső (Dsz, Hungary), Krems-Wachtberg (KW-GH9, Austria), Luochuan (Luo, China) and Fanshan (Fan, China). “Previous separations“ indicate PSDs of old separations published in Újvári et al. (2015).

**Text S3. Reproducibility of Sr, Nd and Hf isotopic analyses**

In order to compare NGRIP dust Sr, Nd and Hf isotopic compositions with PSA samples, repeatability of these analyses must be known. First, samples previously analyzed for Sr-Nd isotopic compositions by Újvári et al. (2015) were size separated and pretreated with acetic acid again and were subjected to Sr-Nd isotope analyses. Figure S3 displays the Sr-Nd isotopic compositions of these five loess samples from SE Asia (Bei, Lin) and Europe (Dsz, Me, Nus). Significant deviations in the ^87^Sr/^86^Sr isotopic ratios, ranging from 0.0032 to 0.0059, can be seen between the old and new separates (Figure S3, panel a), while it is minimal for ^143^Nd/^144^Nd isotope ratios (0.000010–0.000026 or 0.2-0.5 εNd units, Figure S3, panel b). High reproducibility of Nd isotope ratios is also observed for another set of test samples independently prepared for isotopic analyses (Dsz: loess, Hungary; Luo and Luo-RC: loess and red clay from the Luochuan loess sequence, China). A reproducibility of/within 0.2-0.3 εNd units was found for these samples (Figure S4), which is basically the same as for the other sample set. Regarding the Hf isotope compositions, reproducibility was found to be between 0.3-0.8 εHf units, translating to ~0.000009–0.000023 in ^176^Hf/^177^Hf isotopic ratios. This is comparable to the reproducibility of Nd isotope ratios.


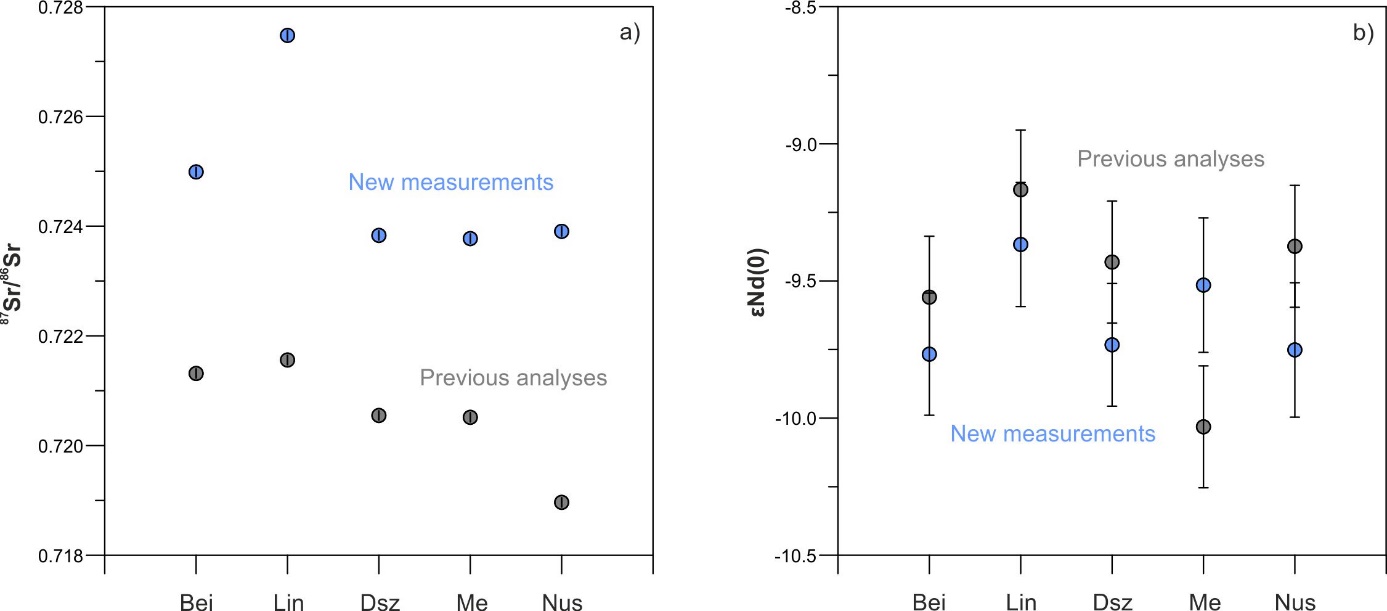


**Figure S3.** Sr and Nd isotopic compositions of the fine fractions of loess samples analyzed in a previous study (Újvári et al., 2015) and re-measured in this study. Note that acetic acid of the same concentration (0.5 mol/L) was used during pretreatments of both sets of samples, but the size separations differed in duration of wet sedimentation. Measurements of old separates shown in gray, new separates in blue. Error bars of individual analyses are smaller than symbols for ^87^Sr/^86^Sr. Sample codes: Bei – Beiguoyuan (CLP, China), Lin – Lingtai (CLP, China), Dsz – Dunaszekcső (Hungary), Me – Mende (Hungary), Nus – Nussloch (Germany).


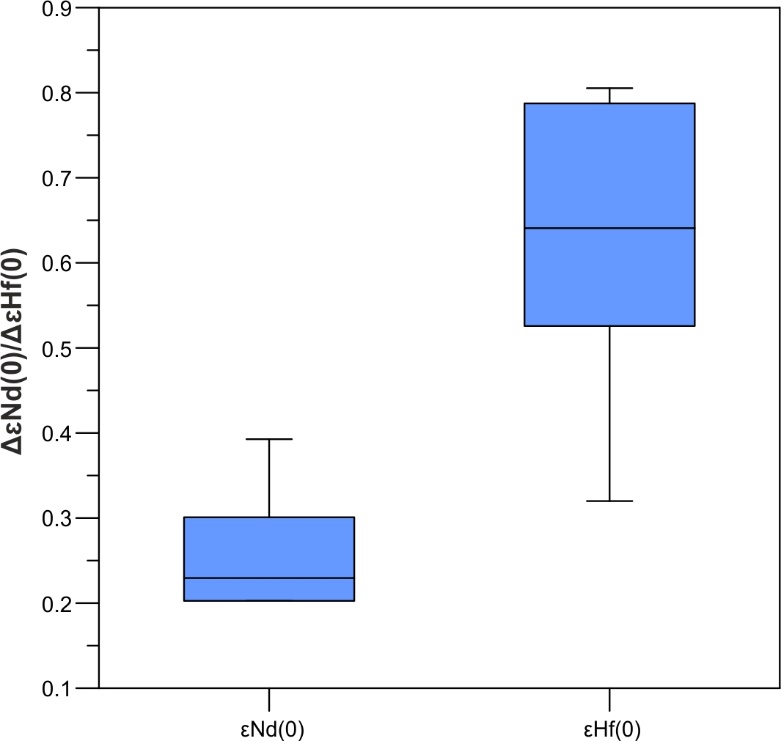


**Figure S4.** Nd and Hf isotopic variability in replicate analyses of the fine fractions (<5, <3 and <2 μm) of loess and red clay samples. The box-whisker plots display differences in εNd(0) and εHf(0) values of the three test samples (Dsz, Luo, Luo-RC) expressed as ΔεNd(0) and ΔεHf(0), calculated as taking the absolute values of the differences of the most radiogenic sub-sample and the other replicates. Nd isotope measurements were undertaken using TIMS, while those of Hf using a Neptune Plus and Nu Plasma II MC-ICPMS. Sample digestions were performed using ammonium-bifluoride at temperatures of 220 and 230 °C.

**Text S4. Additional information on hydrogen (D/H) isotope measurements**

Hydrogen isotope analyses of clay structural water may be compromised by re-adsorption of vapor from ambient air. Figure S5 displays the measured water content of samples as a function of δD values, which are uncorrelated. This demonstrates that the δD values reflect the isotopic composition of the structural OH^–^ groups and are not from contamination (adsorbed, interlayer water), which would otherwise have much more negative δD values.

Hydrogen isotope analyses were undertaken on both the <5 and <2 µm fractions of PSA samples and replicate measurements demonstrate reproducibility of ~2-3 ‰ for both fractions (Figure S6). However, this is larger when the two fractions are compared to each other and varies between 3-9 ‰. This must be considered in any comparison of NGRIP dust δD values with those of PSAs.


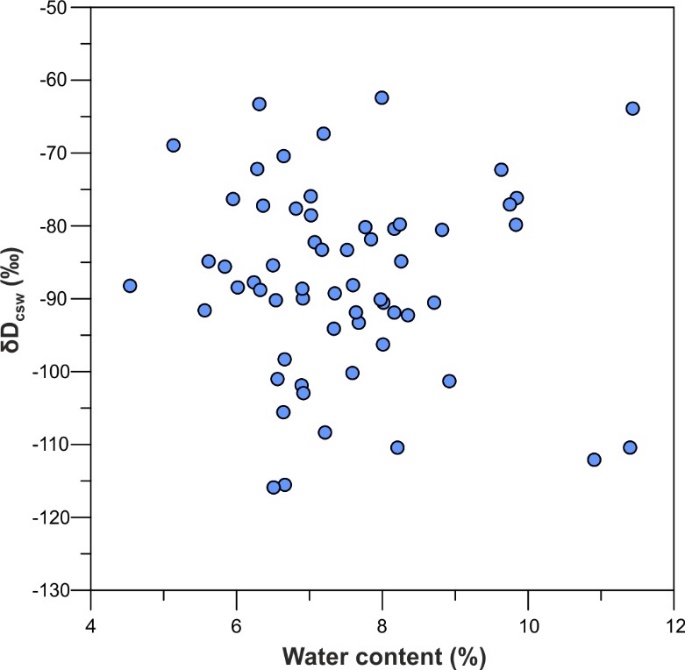


**Figure S5.** The hydrogen isotope composition of clay structural water (δD_csw_, ‰) as a function of water content of the PSA samples and NGRIP dust analyzed in this study. Note that the δD_csw_ data do not reveal any dependency on water content.


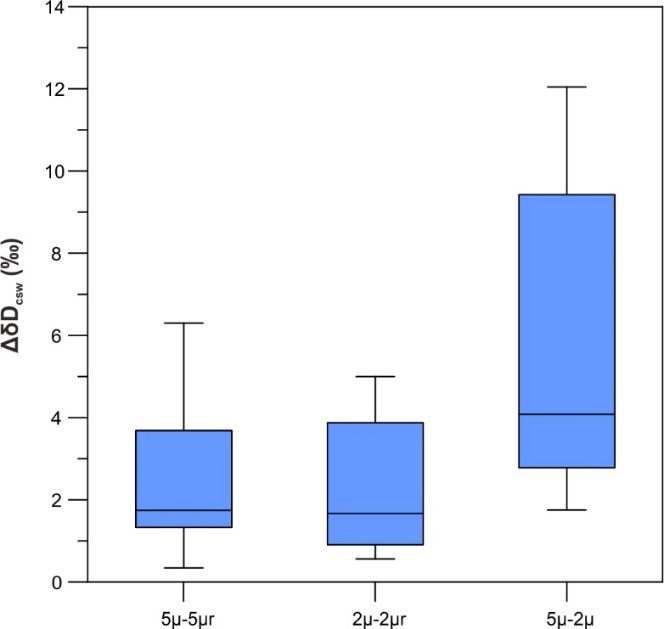


**Figure S6.** Reproducibility of δD_csw_ for replicate measurements on <5 and <2 μm separates (5μ-5μr, 2μ-2μr) and variability of δD_csw_ within <5 and <2 μm separates of the same PSA samples (5μ-2μ). ΔδD_csw_ is the absolute deviation between data pairs, where 5μ-5μr and 2μ-2μr refer to the first and replicate measurements and 5μ-2μ to measurements undertaken on 5μm and 2μm fractions of the same sample. Data displayed include all the PSA samples involved in this study (Supporting Dataset S2).

**Text S5. Additional information to dust transport simulations**

Details of dust transport simulations using the WRF-Chem model are described in the Methods section of the main text. An overview of the used parametrizations and specific settings in WRF-Chem can be found in Tables S1 and S2 below. The monthly distribution of the TOP50/TOP20 events is shown in Table S3. Transit times obtained from the LAGRANTO package (Sprenger and Wernli, 2015) for the TOP20 dust events are given in Table S4. Trajectories and dust load for the TOP20 dust deposition events are depicted in Figures S7.

| **Table S1.** Parametrizations used in the WRF LGM simulation | | |  |
| --- | --- | --- | --- |
| **Parametrization** | **Namelist setting** | **Scheme** | **Reference** |
| Cloud Microphysics | mp_physics = 2 | Purdue Lin | Chen and Sun (2002) |
| Cumulus Physics | cu_physics = 1 | Kain-Fritsch | Kain (2004) |
| Planetary Bounday Layer | bl_pbl_physics = 1 | Yonsei University (YSU) | Hong et al. (2006) |
| Radiation LW | ra_lw_physics = 4 | RRTMG LW | Iacono et al. (2008) |
| Radiation SW | ra_sw_physics = 4 | RRTMG SW | Iacono et al. (2008) |
| Land Surface | sf_surface_physics = 2 | Unified Noah Land surface model | Tewari et al. (2004) |
| Surface Layer | sf_sfclay_phsics = 1 | Revised MM5 | Jimenez et al. (2012) |

| **Table S2.** Specific settings for the WRF LGM simulation (based on PMIP3 21ka experimental design) | | | | | |
| --- | --- | --- | --- | --- | --- |
| **Orbital parameters** | | | **Trace Gas Concentrations** | | |
| Eccentricity | Obliquity | Longitude of Perihelion | CO_2_ | CH_4_ | N_2_O |
| 0.018994 | 22.949° | 294.42° | 185 ppm | 350 ppb | 200 ppb |

| **Table S3.** Number of dust events per month based on TOP20 and TOP50 dust deposition events over Greenland | | | | | | | | | | | | |
| --- | --- | --- | --- | --- | --- | --- | --- | --- | --- | --- | --- | --- |
|  | Jan | Feb | Mar | Apr | May | Jun | Jul | Aug | Sep | Oct | Nov | Dec |
| TOP20 | 2 | 2 | 2 |  | 1 | 1 | 1 | 5 | 3 |  | 1 | 2 |
| TOP50 | 8 | 2 | 5 | 2 | 2 | 1 | 6 | 14 | 4 | 1 | 1 | 4 |

| **Table S4.** Transit times for the TOP20 dust events (in days) | | | |
| --- | --- | --- | --- |
| **Event #** | **Min** | **Mean** | **Max** |
| 1 | 2.88 | 3.63 | 5.00 |
| 2 | 4.00 | 4.90 | 5.75 |
| 3 | 2.62 | 2.80 | 3.12 |
| 4 | 3.75 | 5.27 | 6.25 |
| 5 | 2.50 | 3.21 | 4.25 |
| 6 | 5.38 | 6.14 | 7.75 |
| 7 | 2.00 | 2.42 | 5.00 |
| 8 | 5.88 | 6.98 | 7.38 |
| 9 | 3.00 | 4.73 | 6.00 |
| 10 | 6.75 | 6.88 | 7.12 |
| 11 | 3.50 | 4.14 | 5.00 |
| 12 | 3.75 | 4.04 | 7.12 |
| 13 | 2.62 | 4.43 | 6.75 |
| 14 | 2.88 | 4.29 | 5.00 |
| 15 | 3.12 | 3.32 | 3.50 |
| 16 | 5.25 | 6.41 | 7.50 |
| 17 | 5.88 | 5.88 | 5.88 |
| 18 | 2.88 | 3.90 | 4.25 |
| 19 | 5.50 | 6.28 | 7.00 |
| 20 | 2.88 | 3.97 | 5.62 |
|  |  |  |  |
| Mean | 3.85 | 4.68 | 5.76 |
| SD | 1.40 | 1.37 | 1.36 |

**Figure S7.** Backward trajectories (black lines) for the TOP20 deposition events, atmospheric dust load (shaded, [μg m^-2^ s^-1^], averaged between the time of deposition and time when the first trajectory passed over a region with dust emission) and mean geopotential height field (blue contours, [gpdm]) during the dust transport phase.

**Text S6. Dissimilarity measure calculations**

To quantify similarity/dissimilarity between NGRIP and PSA datasets squared Mahalanobis distances (Md^2^) were calculated. Md^2^ is an effective multivariate distance metric that measures the distance between a data point and a distribution (distribution mean). The Mahalanobis distance is unitless, scale-invariant, and takes into account the correlations of the dataset (De Maesschalck et al., 2000). For calculations of Md^2^ for each data point of ice core dust and PSAs, the clay mineralogical composition (Sme-Ill-Kao-Chl space) and clay mineral ratios (Chl/Kao-Kao/Ill-Sme/Kao) of last glacial dust samples of the GISP2 and GRIP ice core data were random resampled by Monte Carlo simulations (1000 iterations) using the mean and 1SD values of these XRD-based datasets. These MC simulated data (clay mineral compositions and ratios) were used as distributions and the Md^2^ of each data point of the PSA and GISP2 and GRIP dust samples is calculated against the mean of this distribution. To calculate Md^2^ for Sr-Nd-Hf-δD isotope data, the GISP2, GRIP and NGRIP dust sample isotope compositions were used for Sr-Nd (except for sample G2 of the GISP2 ice core, because of potential volcanic contribution), while for Hf and δD the calculations were based on the NGRIP data. The relatively low amount of individual isotope data, especially for Hf and δD, necessitated the generation of a large dataset (in the same way as for clay mineralogy) to which Md^2^ could effectively be calculated. This was done by random sampling of normal distributions defined by the mean and 1SD of the measured isotope data of central Greenland ice cores. Random sampling was performed by Monte Carlo simulations (1000 iterations) and Md^2^ values were subsequently calculated, both in MATLAB environment. Simulated/calculated data are found in Supporting Datasets S1-S2 and Figure S8, while an example MATLAB code for MC simulation of isotope data is found below. Note that MATLAB’s Statistical Toolbox is required for Md^2^ calculations, which was simply performed using the “mahal” function of MATLAB.

Monte Carlo simulation of isotope dataset characteristic for central Greenland (mean and 1SD values were calculated from the GISP2, GRIP and NGRIP data, see “MC_simulations_data” sheet of Supporting Dataset S2)

%MC simulation Sr-Nd-Hf-dD

clear all

% Number of repeats for M-C sim.

i_max = input('Number of steps in M-C simulation: ');

m_Sr = 0.719166;

SD_Sr = 0.000912;

m_Nd = 0.512125;

SD_Nd = 0.000031;

m_Hf = 0.282621;

SD_Hf = 0.000028;

m_dD = -71.31;

SD_dD = 1.25;

%MC simulations

for i = 1: i_max;

Sr = normrnd(m_Sr, SD_Sr);

Nd = normrnd(m_Nd, SD_Nd);

Hf = normrnd(m_Hf, SD_Hf);

dD = normrnd(m_dD, SD_dD);

Sr_out(i,:) = Sr;

Nd_out(i,:) = Nd;

Hf_out(i,:) = Hf;

dD_out(i,:) = dD;

end


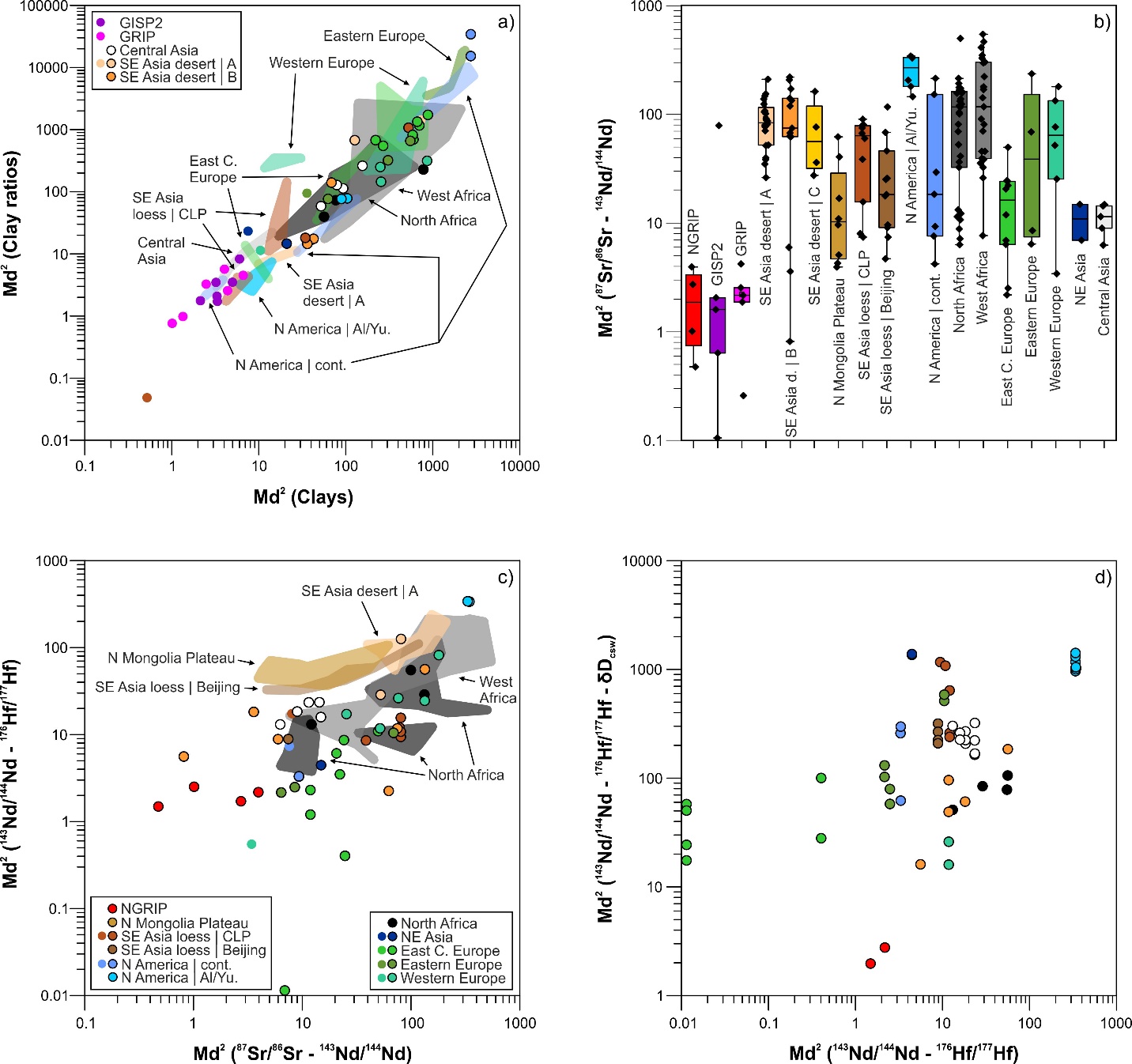


**Figure S8.** Squared Mahalanobis distances (Md^2^) of central Greenland ice core dust and PSA samples calculated from a) clay mineralogical compositions, b) Sr-Nd isotope ratios, c) Sr-Nd and Nd-Hf isotope ratio pairs and d) Nd-Hf and Nd-Hf-δD_csw_ isotope ratios and values. Note that Md^2^ values are also shown for ice core dust samples, providing a basis for the evaluation of Md^2^ values of PSA samples. All of the Md^2^ values were calculated against Monte Carlo simulated compositional distributions (the mean of the distribution) of ice core dust samples from the GISP2, GRIP and NGRIP cores. Md^2^ values calculated from literature data are shown either as fields in panels a) and c) or rimless dots, while new data are denoted by dots with rims.

| **Table S5.** Summary of the likeliness of potential single source contributions to last glacial ice core dust in central Greenland | | | | | | | |
| --- | --- | --- | --- | --- | --- | --- | --- |
| **Source area** | **Clay mineralogy** | **^87^Sr/^86^Sr** | **^143^Nd/^144^Nd** | **^176^Hf/^177^Hf** | **δD_csw_** | **Tracer #** | **Score** |
| Alaska | 2 | 1 | 0 |  |  | 3 | 3 |
| Central Asia | 2 | 1 | 0 | 0 | 0 | 5 | 3 |
| NE Asia / Siberia | 2 | 0 | 2 | 1 | 0 | 5 | 5 |
| SE Asia desert A | 2 | 0 | 0 | 0 |  | 4 | 2 |
| SE Asia desert B | 2 | 1 | 2 | 1 | 1 | 5 | 7 |
| SE Asia desert C | 2 | 0 | 0 |  |  | 3 | 2 |
| Chinese Loess Plateau | 2 | 0 | 2 | 0 | 1 | 5 | 5 |
| Chinese Loess NW Beijing | 1 | 1 | 1 | 0 | 0 | 5 | 3 |
| North Africa | 1 | 1 | 1 | 1 | 1 | 5 | 5 |
| West Africa | 0 | 0 | 0 | 1 |  | 4 | 1 |
| North America - continental | 2 | 0 | 1 | 1 |  | 4 | 4 |
| North America - Nebraska loess | 0 | 1 | 2 | 2 | 0 | 5 | 5 |
| North America - Yukon loess | 1 | 0 | 0 | 0 | 0 | 5 | 1 |
| Eastern Europe | 0 | 1 | 1 | 1 | 0 | 5 | 3 |
| East Central Europe | 1 | 1 | 2 | 2 | 1 | 5 | 7 |
| Western and Central Europe | 1 | 0 | 2 | 0 | 1 | 5 | 4 |
| 0 - incompatible |  |  |  |  |  |  |  |
| 1 - partially compatible |  |  |  |  |  |  |  |
| 2 - compatible |  |  |  |  |  |  |  |

**Text S7. Mixture calculations and modeling**

To clarify if central Greenland dust was a mixture of circum-Pacific volcanic material and SE Asian dust, mixing hyperbola were calculated in Excel using measured elemental concentrations (Sr, Nd, Hf) and ^87^Sr/^86^Sr, ^143^Nd/^144^Nd and ^176^Hf/^177^Hf isotope ratios of the KHG tephra (Kamchatka) and loess (Luo) from the Luochuan loess sequence (Chinese Loess Plateau, CLP, China). Mixing lines were calculated between these two end-members using equations given in Faure and Mensing (2005) and the results are found in Supporting Dataset S2.

Beyond these simple mixing calculations, two and three-component mixing models were established to better understand if 1) mixing of dust from different PSAs in SE Asia (desert region A and B, and CLP dust) and 2) North Africa and Europe (East Central and Western Europe) would explain the isotopic composition of last glacial dust in central Greenland ice cores. First, it was confirmed by normality tests (Kolmogorov-Smirnov and Shapiro-Wilk) that the isotope data of each PSA were significantly drawn from a normally distributed population, and mean and 1SD values were calculated. These mean/1SD values of isotopic compositions, together with elemental concentrations obtained from literature data or our own, unpublished ICP-MS datasets, define normal distributions of end-member compositions (Table S6). Mixing between two and three components (end-members) were Monte Carlo simulated in MATLAB. Fractions of each end-member (F_A_, F_B_, F_C_) were obtained by generating random numbers from continuous uniform distributions, satisfying F_A_ + F_B_ = 1 or F_A_ + F_B_ + F_C_ = 1. The mixing equation of Faure and Mensing (2005)

$R_{M}^{i}= F_{A}R_{A}^{i}\left( {C_{A}^{i}}/{C_{M}^{i}} \right)+F_{B}R_{B}^{i}\left( {C_{B}^{i}}/{C_{M}^{i}} \right)+F_{C}R_{C}^{i}\left( {C_{C}^{i}}/{C_{M}^{i}} \right)$ (Eq. 1)

was calculated with the random generated F_A_, F_B_, F_C_ values and by taking random numbers from the previously defined normally distributed end-member compositions of Sr, Nd or Hf concentrations and ^87^Sr/^86^Sr, ^143^Nd/^144^Nd and ^176^Hf/^177^Hf isotope ratios. In (Eq. 1) R_A_^i^ , R_B_^i^ , R_C_^i^ are the Sr, Nd or Hf isotope ratios, and C_A_^i^ , C_B_^i^ , C_C_^i^ are the elemental concentrations (Sr, Nd or Hf) of end-members A, B or C, while C_M_^i^ and R_M_^i^ are the concentrations and isotope ratios of Sr, Nd or Hf of the two or three-component mixture. For δD, the above equation reduces to

$\delta_{M}^{i}= F_{A}\delta_{A}^{i}+F_{B}\delta_{B}^{i}+F_{C}\delta_{C}^{i}$ (Eq. 2)

, where δ_A_^i^ , δ_B_^i^ , δ_C_^i^ are the δD values of end-members A, B or C and δ_M_^i^ denotes the δD values of the two or three-component mixture.

Two example MATLAB codes for two and three-component mixings are shown below.

% MC SIMULATION / MIXTURE MODELING / TWO COMPONENTS

% SE Asia desert B vs CLP and desert A

clear all

format long g

% Number of repeats for M-C sim.

i_max = input('Number of steps in M-C simulation: ');

% INPUT PARAMETERS OF END-MEMBERS

% SE Asia desert B end-member - component A

m_Sr_A = 285; % mean Sr concentration

SD_Sr_A = 20; % SD of Sr conc.

m_Nd_A = 40; % mean Nd concentration

SD_Nd_A = 10; % SD of Nd conc.

m_Hf_A = 12; % mean Hf concentration

SD_Hf_A = 3; % SD of Hf conc.

m_SrIR_A = 0.726774; % mean Sr isotopic ratio

SD_SrIR_A = 0.004850; % SD of Sr i.r.

m_NdIR_A = 0.512114; % mean Nd i.r.

SD_NdIR_A = 0.000083; % SD of Nd i.r.

m_HfIR_A = 0.282678; % mean Hf i.r.

SD_HfIR_A = 0.000035; % SD of HF i.r.

m_dD_A = -75.3; % mean of dD

SD_dD_A = 9; % SD of dD

% SE Asia desert A end-member - component B

m_Sr_B = 200; % mean Sr concentration

SD_Sr_B = 20; % SD of Sr conc.

m_Nd_B = 30; % mean Nd concentration

SD_Nd_B = 5; % SD of Nd conc.

m_Hf_B = 4; % mean Hf concentration

SD_Hf_B = 1; % SD of Hf conc.

m_SrIR_B = 0.715640; % mean Sr isotopic ratio

SD_SrIR_B = 0.003333; % SD of Sr i.r.

m_NdIR_B = 0.512363; % mean Nd i.r.

SD_NdIR_B = 0.000074; % SD of Nd i.r.

m_HfIR_B = 0.282793; % mean Hf i.r.

SD_HfIR_B = 0.000057; % SD of HF i.r.

% SE Asia loess CLP end-member - component C

m_Sr_C = 120; % mean Sr concentration

SD_Sr_C = 15; % SD of Sr conc.

m_Nd_C = 30; % mean Nd concentration

SD_Nd_C = 5; % SD of Nd conc.

m_Hf_C = 3.5; % mean Hf concentration

SD_Hf_C = 0.5; % SD of Hf conc.

m_SrIR_C = 0.725343; % mean Sr isotopic ratio

SD_SrIR_C = 0.002435; % SD of Sr i.r.

m_NdIR_C = 0.512141; % mean Nd i.r.

SD_NdIR_C = 0.000066; % SD of Nd i.r.

m_HfIR_C = 0.282733; % mean Hf i.r.

SD_HfIR_C = 0.000030; % SD of HF i.r.

m_dD_C = -93.1; % mean of dD

SD_dD_C = 16; % SD of dD

% MC simulations

for i = 1: i_max;

FA = unifrnd(0,1); % fraction of component A (values: 0-1)

FA_out(i,:) = FA;

FB = 1-FA; % fraction of component B

FB_out(i,:) = FB;

% simulations of Sr isotope ratios for A-B and A-C

r_Sr_A = normrnd(m_Sr_A,SD_Sr_A);

r_Sr_B = normrnd(m_Sr_B,SD_Sr_B);

r_Sr_C = normrnd(m_Sr_C,SD_Sr_C);

Sr_M_AB = (FA*r_Sr_A)+(FB*r_Sr_B);

Sr_M_AC = (FA*r_Sr_A)+(FB*r_Sr_C);

Sr_A = r_Sr_A/Sr_M_AB;

Sr_B = r_Sr_B/Sr_M_AB;

Sr_A_ = r_Sr_A/Sr_M_AC;

Sr_C = r_Sr_C/Sr_M_AC;

r_SrIR_A = normrnd(m_SrIR_A,SD_SrIR_A);

r_SrIR_B = normrnd(m_SrIR_B,SD_SrIR_B);

r_SrIR_C = normrnd(m_SrIR_C,SD_SrIR_C);

r_SrIR_A_out(i,:) = r_SrIR_A;

r_SrIR_B_out(i,:) = r_SrIR_B;

r_SrIR_C_out(i,:) = r_SrIR_C;

SrIC_AB = (FA*r_SrIR_A*Sr_A)+(FB*r_SrIR_B*Sr_B);

SrIC_AC = (FA*r_SrIR_A*Sr_A_)+(FB*r_SrIR_C*Sr_C);

SrIC_AB_out(i,:) = SrIC_AB;

SrIC_AC_out(i,:) = SrIC_AC;

% simulations of Nd isotope ratios for A-B and A-C

r_Nd_A = normrnd(m_Nd_A,SD_Nd_A);

r_Nd_B = normrnd(m_Nd_B,SD_Nd_B);

r_Nd_C = normrnd(m_Nd_C,SD_Nd_C);

Nd_M_AB = (FA*r_Nd_A)+(FB*r_Nd_B);

Nd_M_AC = (FA*r_Nd_A)+(FB*r_Nd_C);

Nd_A = r_Nd_A/Nd_M_AB;

Nd_B = r_Nd_B/Nd_M_AB;

Nd_A_ = r_Nd_A/Nd_M_AC;

Nd_C = r_Nd_C/Nd_M_AC;

r_NdIR_A = normrnd(m_NdIR_A,SD_NdIR_A);

r_NdIR_B = normrnd(m_NdIR_B,SD_NdIR_B);

r_NdIR_C = normrnd(m_NdIR_C,SD_NdIR_C);

r_eNd_A = ((r_NdIR_A/0.51263)-1)*10000;

r_eNd_B = ((r_NdIR_B/0.51263)-1)*10000;

r_eNd_C = ((r_NdIR_C/0.51263)-1)*10000;

r_eNd_A_out(i,:) = r_eNd_A;

r_eNd_B_out(i,:) = r_eNd_B;

r_eNd_C_out(i,:) = r_eNd_C;

NdIC_AB = (FA*r_NdIR_A*Nd_A)+(FB*r_NdIR_B*Nd_B);

NdIC_AC = (FA*r_NdIR_A*Nd_A_)+(FB*r_NdIR_C*Nd_C);

NdIC_AB_out(i,:) = NdIC_AB;

NdIC_AC_out(i,:) = NdIC_AC;

eNd_AB = ((NdIC_AB/0.51263)-1)*10000;

eNd_AB_out(i,:) = eNd_AB;

eNd_AC = ((NdIC_AC/0.51263)-1)*10000;

eNd_AC_out(i,:) = eNd_AC;

% simulations of Hf isotope ratios for A-B and A-C

r_Hf_A = normrnd(m_Hf_A,SD_Hf_A);

r_Hf_B = normrnd(m_Hf_B,SD_Hf_B);

r_Hf_C = normrnd(m_Hf_C,SD_Hf_C);

Hf_M_AB = (FA*r_Hf_A)+(FB*r_Hf_B);

Hf_M_AC = (FA*r_Hf_A)+(FB*r_Hf_C);

Hf_A = r_Hf_A/Hf_M_AB;

Hf_B = r_Hf_B/Hf_M_AB;

Hf_A_ = r_Hf_A/Hf_M_AC;

Hf_C = r_Hf_C/Hf_M_AC;

r_HfIR_A = normrnd(m_HfIR_A,SD_HfIR_A);

r_HfIR_B = normrnd(m_HfIR_B,SD_HfIR_B);

r_HfIR_C = normrnd(m_HfIR_C,SD_HfIR_C);

r_eHf_A = ((r_HfIR_A/0.282785)-1)*10000;

r_eHf_B = ((r_HfIR_B/0.282785)-1)*10000;

r_eHf_C = ((r_HfIR_C/0.282785)-1)*10000;

r_eHf_A_out(i,:) = r_eHf_A;

r_eHf_B_out(i,:) = r_eHf_B;

r_eHf_C_out(i,:) = r_eHf_C;

HfIC_AB = (FA*r_HfIR_A*Hf_A)+(FB*r_HfIR_B*Hf_B);

HfIC_AC = (FA*r_HfIR_A*Hf_A_)+(FB*r_HfIR_C*Hf_C);

HfIC_AB_out(i,:) = HfIC_AB;

HfIC_AC_out(i,:) = HfIC_AC;

eHf_AB = ((HfIC_AB/0.282785)-1)*10000;

eHf_AB_out(i,:) = eHf_AB;

eHf_AC = ((HfIC_AC/0.282785)-1)*10000;

eHf_AC_out(i,:) = eHf_AC;

% simulations of dD values for A-B and A-C

r_dD_A = normrnd(m_dD_A,SD_dD_A);

r_dD_C = normrnd(m_dD_C,SD_dD_C);

r_dD_A_out(i,:) = r_dD_A;

r_dD_C_out(i,:) = r_dD_C;

dD_AC = (FA*r_dD_A) + (FB*r_dD_C);

dD_AC_out(i,:) = dD_AC;

end

% MC SIMULATION / MIXTURE MODELING / THREE COMPONENTS

% North Africa vs EC and W Europe

clear all

format long g

% Number of repeats for M-C sim.

i_max = input('Number of steps in M-C simulation: ');

% INPUT PARAMETERS OF END-MEMBERS

% North African end-member - component A

m_Sr_A = 180; % mean Sr concentration

SD_Sr_A = 10; % SD of Sr conc.

m_Nd_A = 50; % mean Nd concentration

SD_Nd_A = 5; % SD of Nd conc.

m_Hf_A = 3.8; % mean Hf concentration

SD_Hf_A = 0.5; % SD of Hf conc.

m_SrIR_A = 0.724617; % mean Sr isotopic ratio

SD_SrIR_A = 0.004504; % SD of Sr i.r.

m_NdIR_A = 0.512006; % mean Nd i.r.

SD_NdIR_A = 0.000051; % SD of Nd i.r.

m_HfIR_A = 0.282659; % mean Hf i.r.

SD_HfIR_A = 0.000035; % SD of HF i.r.

m_dD_A = -63.2; % mean of dD

SD_dD_A = 1; % SD of dD

% East Central Europe end-member - component B

m_Sr_B = 110; % mean Sr concentration

SD_Sr_B = 20; % SD of Sr conc.

m_Nd_B = 28; % mean Nd concentration

SD_Nd_B = 5; % SD of Nd conc.

m_Hf_B = 3; % mean Hf concentration

SD_Hf_B = 0.5; % SD of Hf conc.

m_SrIR_B = 0.723478; % mean Sr isotopic ratio

SD_SrIR_B = 0.001310; % SD of Sr i.r.

m_NdIR_B = 0.512137; % mean Nd i.r.

SD_NdIR_B = 0.000009; % SD of Nd i.r.

m_HfIR_B = 0.282593; % mean Hf i.r.

SD_HfIR_B = 0.000042; % SD of HF i.r.

m_dD_B = -79.1; % mean of dD

SD_dD_B = 3; % SD of dD

% Western Europe end-member - component C

m_Sr_C = 130; % mean Sr concentration

SD_Sr_C = 30; % SD of Sr conc.

m_Nd_C = 30; % mean Nd concentration

SD_Nd_C = 5; % SD of Nd conc.

m_Hf_C = 4; % mean Hf concentration

SD_Hf_C = 1; % SD of Hf conc.

m_SrIR_C = 0.727587; % mean Sr isotopic ratio

SD_SrIR_C = 0.003055; % SD of Sr i.r.

m_NdIR_C = 0.512081; % mean Nd i.r.

SD_NdIR_C = 0.000031; % SD of Nd i.r.

m_HfIR_C = 0.282482; % mean Hf i.r.

SD_HfIR_C = 0.000059; % SD of HF i.r.

m_dD_C = -72.4; % mean of dD

SD_dD_C = 5; % SD of dD

% MC simulations

for i = 1: i_max;

FA = unifrnd(0,1); % fraction of component A (values: 0-1)

FA_out(i,:) = FA;

Fs = 1-FA;

FB = unifrnd(0,Fs); % fraction of component B

FB_out(i,:) = FB;

FC = 1-FA-FB; % fraction of component C

FC_out(i,:) = FC;

% simulations of Sr isotope ratios for comp. A-B-C

r_Sr_A = normrnd(m_Sr_A,SD_Sr_A);

r_Sr_B = normrnd(m_Sr_B,SD_Sr_B);

r_Sr_C = normrnd(m_Sr_C,SD_Sr_C);

Sr_M = (FA*r_Sr_A)+(FB*r_Sr_B)+(FC*r_Sr_C);

Sr_A = r_Sr_A/Sr_M;

Sr_B = r_Sr_B/Sr_M;

Sr_C = r_Sr_C/Sr_M;

r_SrIR_A = normrnd(m_SrIR_A,SD_SrIR_A);

r_SrIR_B = normrnd(m_SrIR_B,SD_SrIR_B);

r_SrIR_C = normrnd(m_SrIR_C,SD_SrIR_C);

r_SrIR_A_out(i,:) = r_SrIR_A;

r_SrIR_B_out(i,:) = r_SrIR_B;

r_SrIR_C_out(i,:) = r_SrIR_C;

SrIC_mix = (FA*r_SrIR_A*Sr_A)+(FB*r_SrIR_B*Sr_B)+(FC*r_SrIR_C*Sr_C);

SrIC_mix_out(i,:) = SrIC_mix;

% simulations of Nd isotope ratios for comp. A-B-C

r_Nd_A = normrnd(m_Nd_A,SD_Nd_A);

r_Nd_B = normrnd(m_Nd_B,SD_Nd_B);

r_Nd_C = normrnd(m_Nd_C,SD_Nd_C);

Nd_M = (FA*r_Nd_A)+(FB*r_Nd_B)+(FC*r_Nd_C);

Nd_A = r_Nd_A/Nd_M;

Nd_B = r_Nd_B/Nd_M;

Nd_C = r_Nd_C/Nd_M;

r_NdIR_A = normrnd(m_NdIR_A,SD_NdIR_A);

r_NdIR_B = normrnd(m_NdIR_B,SD_NdIR_B);

r_NdIR_C = normrnd(m_NdIR_C,SD_NdIR_C);

r_eNd_A = ((r_NdIR_A/0.51263)-1)*10000;

r_eNd_B = ((r_NdIR_B/0.51263)-1)*10000;

r_eNd_C = ((r_NdIR_C/0.51263)-1)*10000;

r_eNd_A_out(i,:) = r_eNd_A;

r_eNd_B_out(i,:) = r_eNd_B;

r_eNd_C_out(i,:) = r_eNd_C;

NdIC_mix = (FA*r_NdIR_A*Nd_A)+(FB*r_NdIR_B*Nd_B)+(FC*r_NdIR_C*Nd_C);

NdIC_mix_out(i,:) = NdIC_mix;

eNd_mix = ((NdIC_mix/0.51263)-1)*10000;

eNd_mix_out(i,:) = eNd_mix;

% simulations of Hf isotope ratios for comp. A-B-C

r_Hf_A = normrnd(m_Hf_A,SD_Hf_A);

r_Hf_B = normrnd(m_Hf_B,SD_Hf_B);

r_Hf_C = normrnd(m_Hf_C,SD_Hf_C);

Hf_M = (FA*r_Hf_A)+(FB*r_Hf_B)+(FC*r_Hf_C);

Hf_A = r_Hf_A/Hf_M;

Hf_B = r_Hf_B/Hf_M;

Hf_C = r_Hf_C/Hf_M;

r_HfIR_A = normrnd(m_HfIR_A,SD_HfIR_A);

r_HfIR_B = normrnd(m_HfIR_B,SD_HfIR_B);

r_HfIR_C = normrnd(m_HfIR_C,SD_HfIR_C);

r_eHf_A = ((r_HfIR_A/0.282785)-1)*10000;

r_eHf_B = ((r_HfIR_B/0.282785)-1)*10000;

r_eHf_C = ((r_HfIR_C/0.282785)-1)*10000;

r_eHf_A_out(i,:) = r_eHf_A;

r_eHf_B_out(i,:) = r_eHf_B;

r_eHf_C_out(i,:) = r_eHf_C;

HfIC_mix = (FA*r_HfIR_A*Hf_A)+(FB*r_HfIR_B*Hf_B)+(FC*r_HfIR_C*Hf_C);

HfIC_mix_out(i,:) = HfIC_mix;

eHf_mix = ((HfIC_mix/0.282785)-1)*10000;

eHf_mix_out(i,:) = eHf_mix;

% simulations of dD values for comp. A-B-C

r_dD_A = normrnd(m_dD_A,SD_dD_A);

r_dD_B = normrnd(m_dD_B,SD_dD_B);

r_dD_C = normrnd(m_dD_C,SD_dD_C);

r_dD_A_out(i,:) = r_dD_A;

r_dD_B_out(i,:) = r_dD_B;

r_dD_C_out(i,:) = r_dD_C;

dD_mix = (FA*r_dD_A)+(FB*r_dD_B)+(FC*r_dD_C);

dD_mix_out(i,:) = dD_mix;

end

| **Table S6.** End-member compositions | | | |  |  |  |  |  |  |  |  |  |  |  |  |
| --- | --- | --- | --- | --- | --- | --- | --- | --- | --- | --- | --- | --- | --- | --- | --- |
| **EM** | **Sr** | **1SD** | **Nd** | **1SD** | **Hf** | **1SD** | **^87^Sr/^86^Sr** | **1SD** | **^143^Nd/^144^Nd** | **1SD** | **^176^Hf/^177^Hf** | **1SD** | **δD_csw_ (‰)** | **1SD** | **Source of elemental concentrations** |
| Central Asia | 130 | 15 | 25 | 5 | 3.5 | 0.5 | 0.718967 | 0.002056 | 0.512209 | 0.000011 | 0.282710 | 0.000017 | -88.4 | 2 | Yang et al. (2006) |
| North Africa | 180 | 10 | 50 | 5 | 3.8 | 0.5 | 0.724617 | 0.004504 | 0.512006 | 0.000051 | 0.282659 | 0.000035 | -63.2 | 1 | Muhs et al. (2007), Pourmand et al. (2014) |
| North America - Nebraska | 75 | 10 | 22 | 5 | 2.5 | 0.5 | 0.717998 | 0.002576 | 0.512099 | 0.000043 | 0.282628 | 0.000015 | -91.1 | 5.5 | unpublished ICP-MS data |
| North America - Yukon | 250 | 50 | 15 | 5 | 3 | 0.5 | 0.710072 | 0.000399 | 0.512602 | 0.000010 | 0.282895 | 0.000003 | -105 | 3.8 | George et al. (2003) |
| East Central Europe | 110 | 20 | 28 | 5 | 3 | 0.5 | 0.723478 | 0.001310 | 0.512137 | 0.000009 | 0.282593 | 0.000042 | -79.1 | 3 | unpublished ICP-MS data |
| Western Europe | 130 | 30 | 30 | 5 | 4 | 1 | 0.727587 | 0.003055 | 0.512081 | 0.000031 | 0.282482 | 0.000059 | -72.4 | 5 | unpublished data; Chauvel et al. (2014); Bosq et al. (2020) |
| SE Asia desert A | 200 | 20 | 30 | 5 | 4 | 1 | 0.715640 | 0.003333 | 0.512363 | 0.000074 | 0.282793 | 0.000057 |  |  | Jeong (2020) |
| SE Asia desert B | 285 | 20 | 40 | 10 | 12 | 3 | 0.726774 | 0.004850 | 0.512114 | 0.000083 | 0.282678 | 0.000035 | -75.3 | 9 | Chang et al. (2000); Honda et al., (2004); Yang et al. (2007); Ferrat et al. (2011) |
| SE Asia loess CLP | 120 | 15 | 30 | 5 | 3.5 | 0.5 | 0.725343 | 0.002435 | 0.512141 | 0.000066 | 0.282733 | 0.000030 | -93.1 | 16 | Yokoo et al. (2004); Feng et al. (2009); Hao et al. (2010); Ferrat et al. (2011); unpublished ICP-MS data |

Results of mixture modeling between two or three end-members are shown in Figure S9-S13 and given in Supporting Dataset S3.


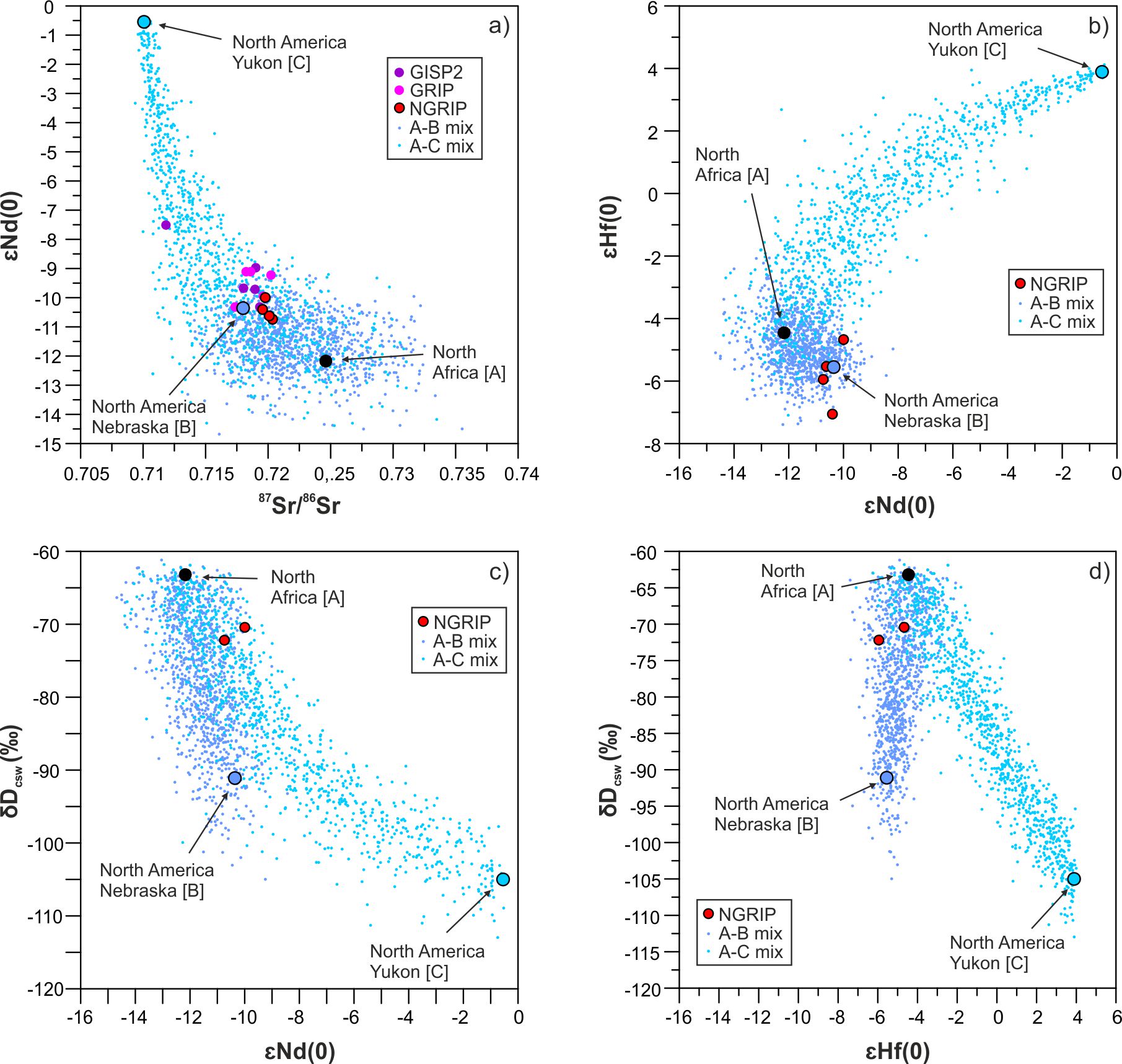


**Figure S9.** Simulated two-component mixtures between end-members of North Africa and North America (Nebraska and Yukon loess) with central Greenland ice core dust compositions. For end-member compositions see Table S6. Note that dots representing end-members only indicate the mean of normal distributions (which formed the basis of MC simulations) and these distributions are not shown. MC iterations: 1000.


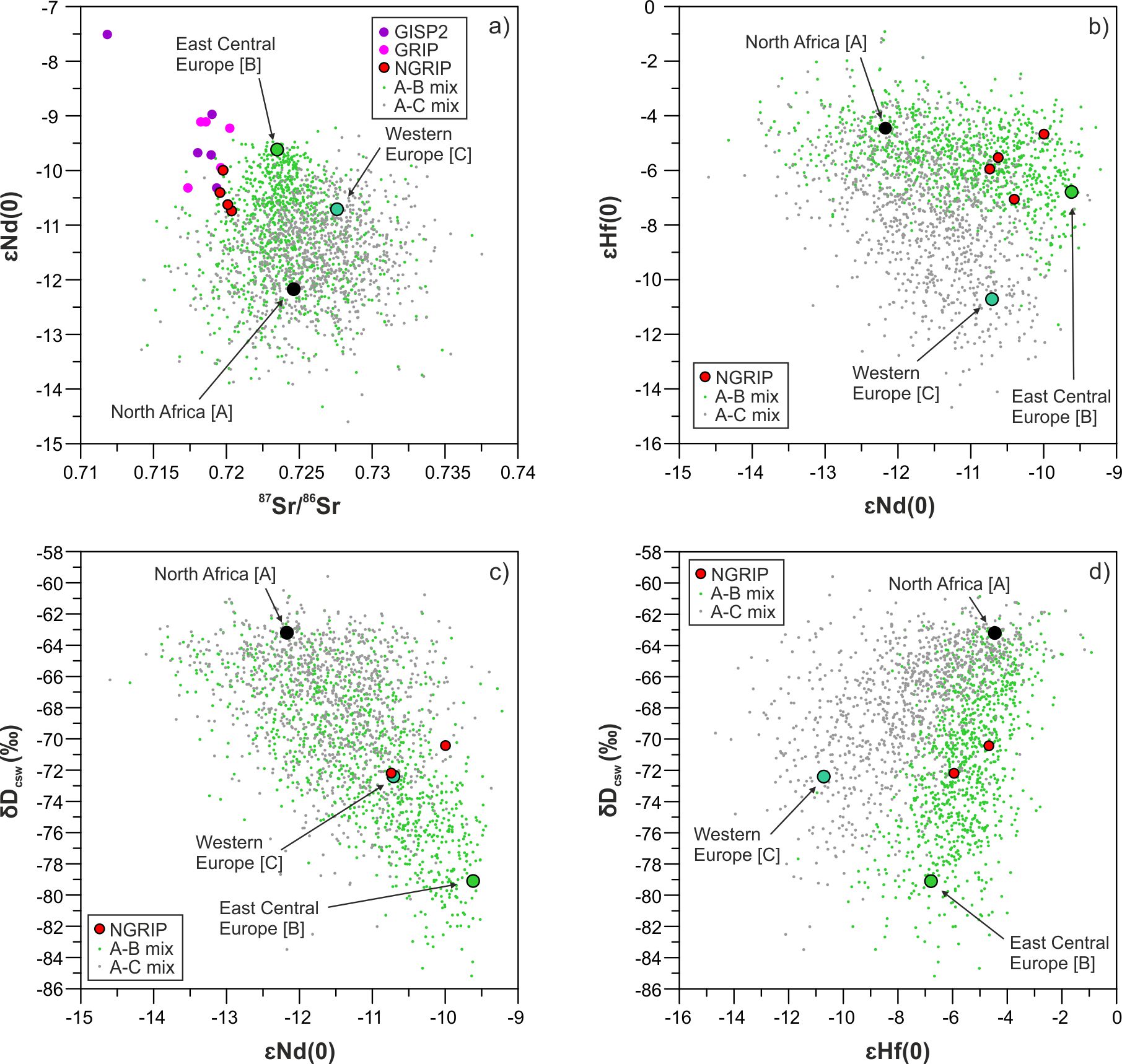


**Figure S10.** Simulated two-component mixtures between end-members of North Africa and East Central/Western Europe with central Greenland ice core dust compositions. For end-member compositions see Table S6. Note that dots representing end-members only indicate the mean of normal distributions (which formed the basis of MC simulations) and these distributions are not shown. MC iterations: 1000.


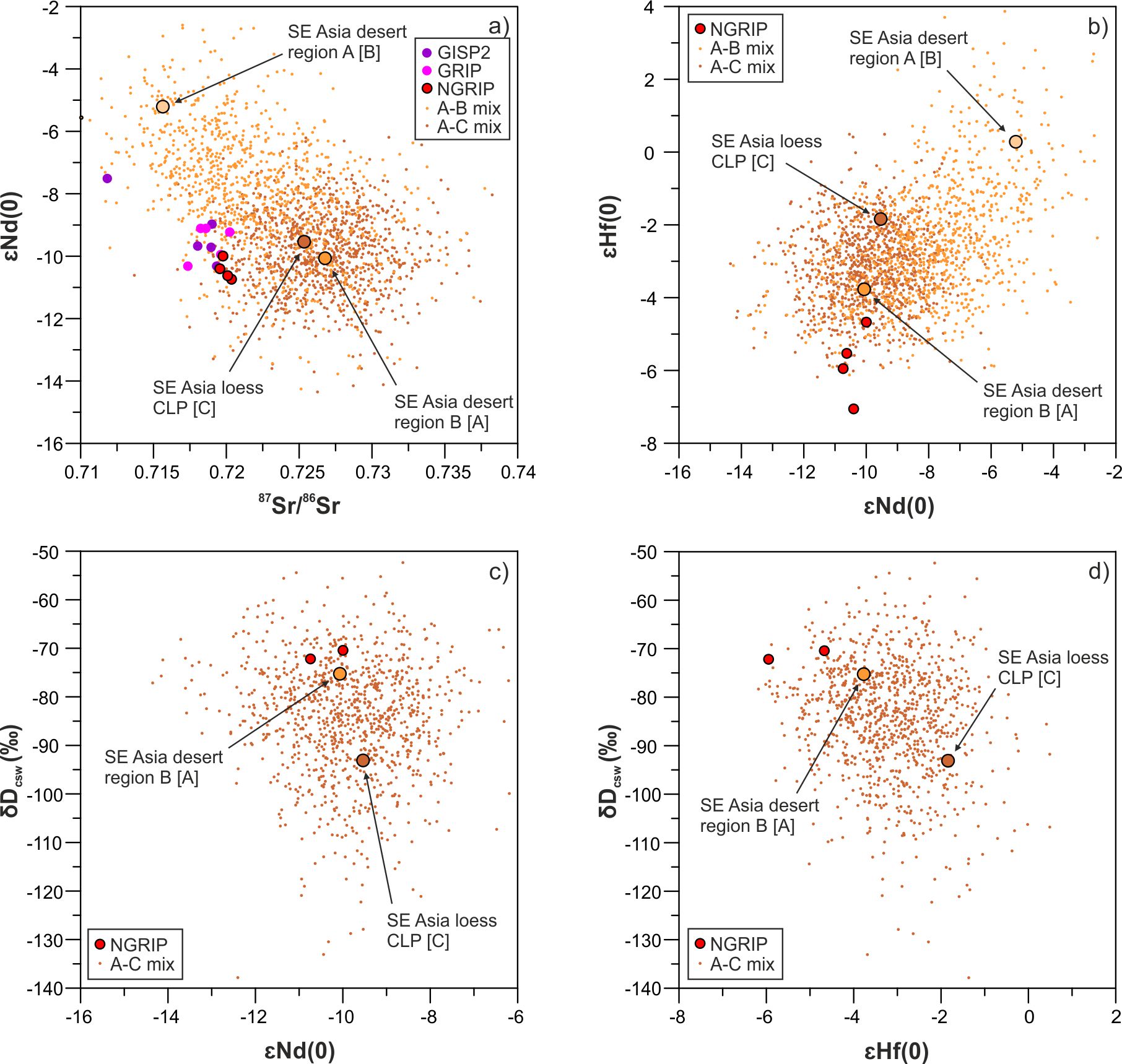


**Figure S11.** Simulated two-component mixtures between end-members of SE Asia with central Greenland ice core dust compositions. For end-member compositions see Table S6. Note that dots representing end-members only indicate the mean of normal distributions (which formed the basis of MC simulations) and these distributions are not shown. MC iterations: 1000.


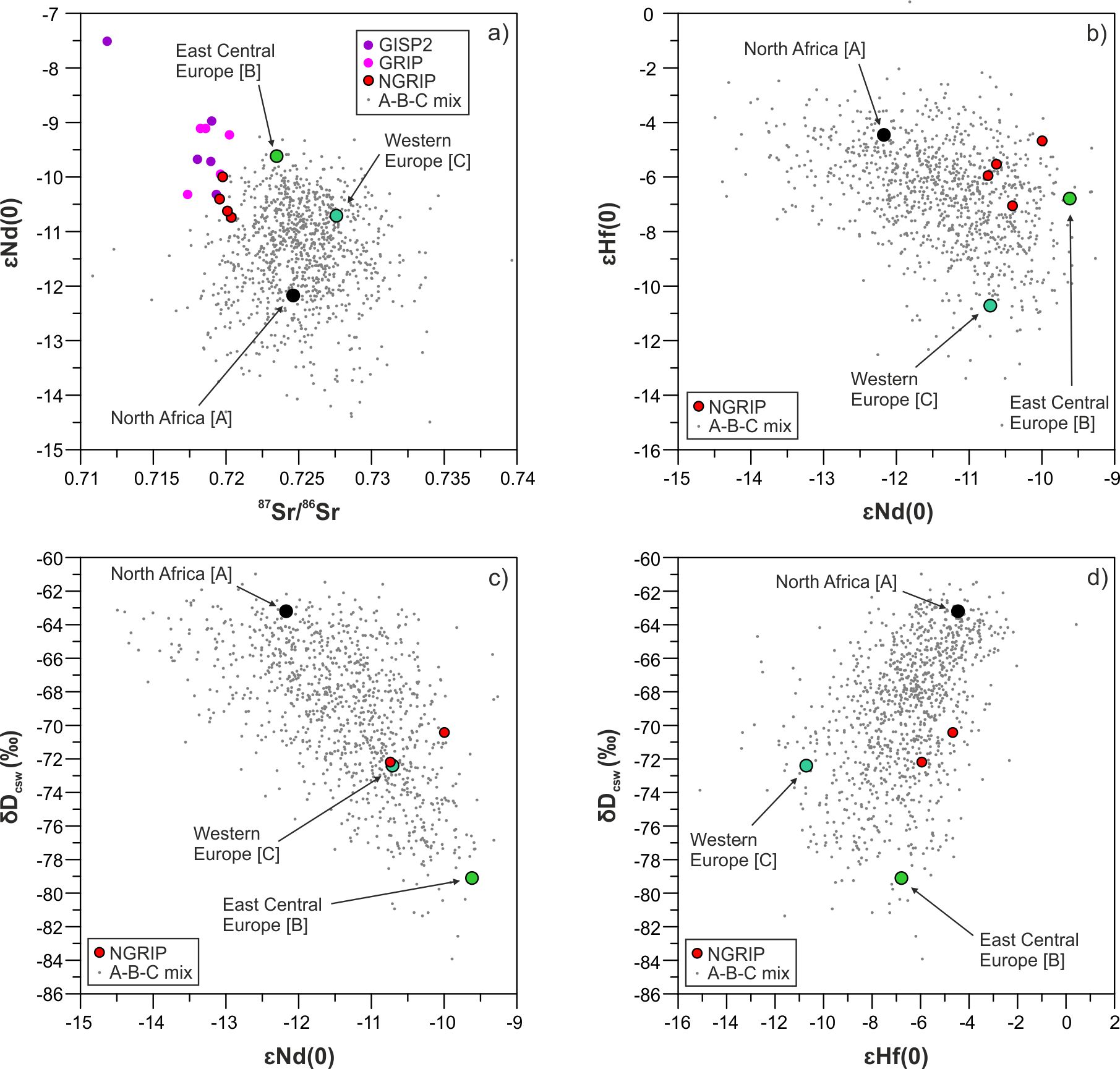


**Figure S12.** Simulated three-component mixtures between end-members of North Africa and East Central/Western Europe with central Greenland ice core dust compositions. For end-member compositions see Table S6. Note that dots representing end-members only indicate the mean of normal distributions (which formed the basis of MC simulations) and these distributions are not shown. MC iterations: 1000.


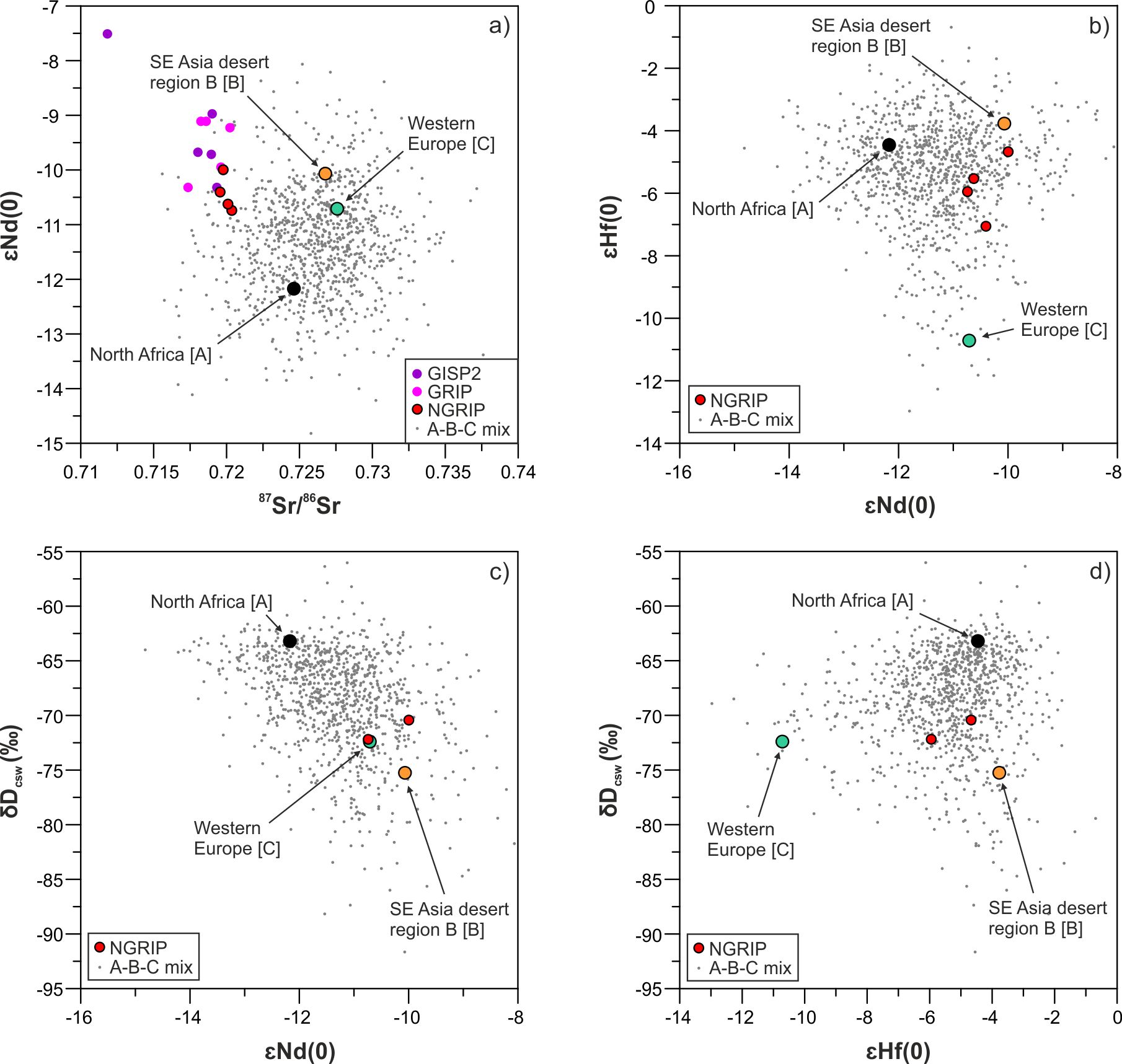


**Figure S13.** Simulated three-component mixtures between end-members of North Africa, Western Europe and SE Asia desert region B with central Greenland ice core dust compositions. For end-member compositions see Table S6. Note that dots representing end-members only indicate the mean of normal distributions (which formed the basis of MC simulations) and these distributions are not shown. MC iterations: 1000.

| **Table S7.** Isotopic compositions of simulated North African and North American dust mixtures broadly matching the isotopic compositions of central Greenland last glacial dust (out of 1000 MC iterations displayed in Figure S9) | | | | | | | | | | | | | | | | | | | |
| --- | --- | --- | --- | --- | --- | --- | --- | --- | --- | --- | --- | --- | --- | --- | --- | --- | --- | --- | --- |
| **Source area** | **F_A_** | | **F_B-C_** | | **^87^Sr/^86^Sr** | | **^143^Nd/^144^Nd** | | **εNd(0)** | | **^176^Hf/^177^Hf** | | **εHf(0)** | | **δD_csw_** | | **Match score** | |  |
| continental US / Nebraska | 0.93 | | 0.07 | | 0.717489 | | 0.512110 | | -10.15 | | 0.282659 | | -4.46 | | -65.2 | | 1 | |  |
|  | 0.45 | | 0.55 | | 0.718407 | | 0.512074 | | -10.85 | | 0.282656 | | -4.55 | | -77.3 | | 2 | |  |
|  | 0.15 | | 0.85 | | 0.719092 | | 0.512073 | | -10.87 | | 0.282599 | | -6.59 | | -77.9 | | 2 | |  |
|  | 0.44 | | 0.56 | | 0.719251 | | 0.512112 | | -10.11 | | 0.282641 | | -5.09 | | -79.3 | | 2 | |  |
|  | 0.55 | | 0.45 | | 0.719276 | | 0.512079 | | -10.76 | | 0.282661 | | -4.40 | | -76.7 | | 2 | |  |
|  | 0.18 | | 0.82 | | 0.719516 | | 0.512072 | | -10.89 | | 0.282655 | | -4.59 | | -75.8 | | 3 | |  |
|  | 0.90 | | 0.10 | | 0.719774 | | 0.512073 | | -10.87 | | 0.282665 | | -4.25 | | -65.0 | | 1 | |  |
|  | 0.36 | | 0.64 | | 0.719816 | | 0.512085 | | -10.63 | | 0.282629 | | -5.50 | | -79.9 | | 2 | |  |
|  | 0.58 | | 0.42 | | 0.719835 | | 0.512114 | | -10.06 | | 0.282665 | | -4.23 | | -75.6 | | 3 | |  |
|  | 0.49 | | 0.51 | | 0.720254 | | 0.512089 | | -10.55 | | 0.282636 | | -5.26 | | -78.7 | | 2 | |  |
| Yukon | 0.66 | | 0.34 | | 0.717994 | | 0.512098 | | -10.39 | | 0.282660 | | -4.42 | | -77.9 | | 1 | |  |
|  | 0.77 | | 0.23 | | 0.719546 | | 0.512076 | | -10.81 | | 0.282657 | | -4.51 | | -73.7 | | 3 | |  |
|  |  | |  | |  | |  | |  | |  | |  | |  | |  | |  |
| Mean of LGM ice core dust (GISP2, GRIP) |  | |  | | 0.718820 | | 0.512138 | | -9.60 | |  | |  | |  | |  | |  |
| Mean of LGM ice core dust (NGRIP) |  | |  | | 0.719945 | | 0.512095 | | -10.44 | | 0.282621 | | -5.80 | | -71.3 | |  | |  |
| F_A_ and F_B-C_ are the random simulated end-member fractions in mixtures (F_A_ - North Africa, F_B_ - Nebraska loess, F_C_ - Yukon loess) | | | | | | | | | | | | | | | | | | | |
| Match score is estimated against last glacial Greenland ice core dust isotope compositions: 1 - low, 2 - fair, 3 - high | | | | | | | | | | | | | | | | | |  | |
| Filtering of the original MC simulation dataset (displayed in Figure S9) was done using the isotopic range | | | | | | | | | | | | | | | |  | |  | |
| of LGM ice core dust (GISP2, GRIP and NGRIP), considering measurement reproducibilities: | | | | | | | | | | | | | |  | |  | |  | |
| δD_csw_: -80.3 to -62.3 permil | |  | |  | |  | |  | |  | |  | |  | |  | |  | |
| εHf(0): -7.57 to -4.16 | |  | |  | |  | |  | |  | |  | |  | |  | |  | |
| εNd(0): -10.97 to -8.74 | |  | |  | |  | |  | |  | |  | |  | |  | |  | |
| ^87^Sr/^86^Sr: 0.720387 to 0.717330 | |  | |  | |  | |  | |  | |  | |  | |  | |  | |


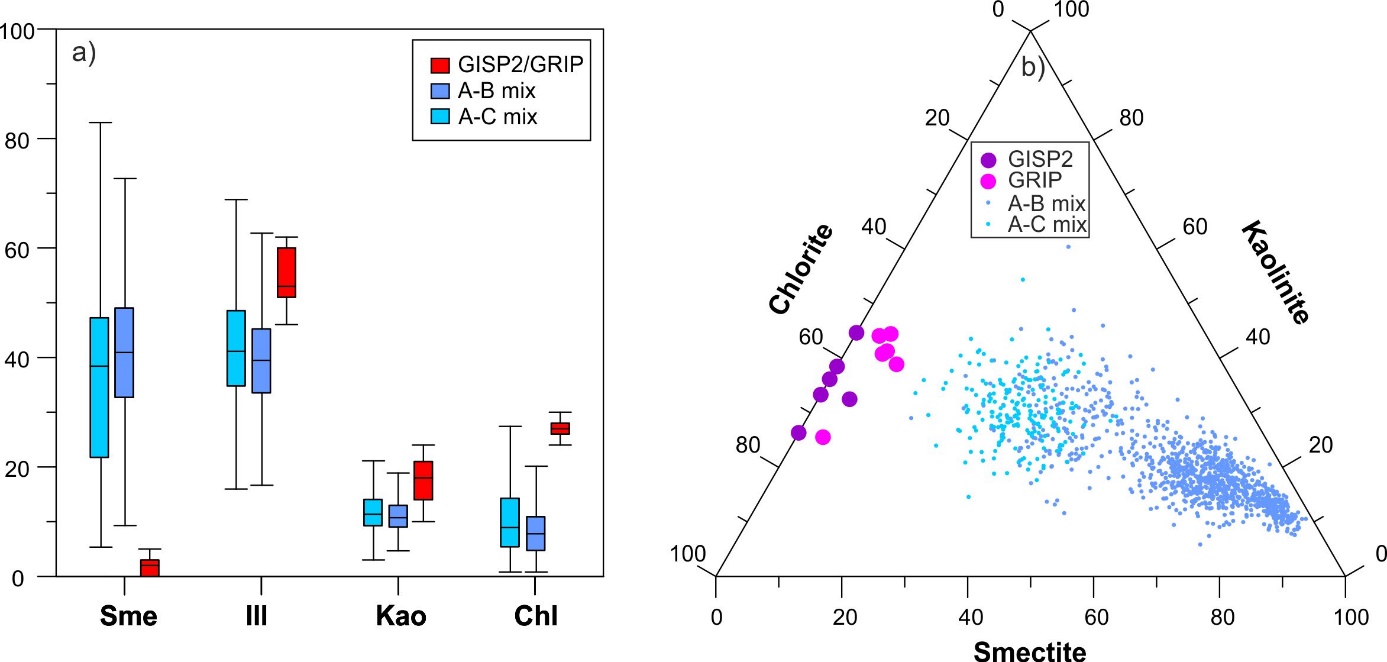


**Figure S14.** Box-whisker a) and ternary b) diagrams of simulated clay mineralogy of two component North African (A) and North American (Nebraska and Yukon loess – B, C) dust mixtures using F_A_ and F_B-C_ end-member fractions given in Table S7. GISP2 and GRIP ice core dust compositions are also shown for comparison. Random sampling of clay mineralogy distributions was undertaken in 100 MC iterations for each F_A_ and F_B-C_ pairs given in Table S7 and thus simulation data displayed here include 1200 data points. The clay mineralogy distributions were defined from the XRD dataset given in Supporting Dataset S1 and were as follows: North African dust – smectite: 17±11 (mean±2SD), illite: 53±19, kaolinite: 15±8.6, chlorite: 16±11; North America / Nebraska - smectite: 64.2±19.8, illite: 27.6±16.8, kaolinite: 7.4±2.7, chlorite: 0.8±0.6; North America / Yukon - smectite: 15.5±0.7, illite: 44±1.4, kaolinite: 15.5±0.7, chlorite: 25±1. More information in the main text.

| **Table S8.** Isotopic compositions of simulated North African and European dust mixtures matching the isotopic compositions of central Greenland last glacial dust (out of 1000 MC iterations displayed in Figure S10) | | | | | | | | | |
| --- | --- | --- | --- | --- | --- | --- | --- | --- | --- |
| **Source area** | **F_A_** | **F_B-C_** | **^87^Sr/^86^Sr** | **^143^Nd/^144^Nd** | **εNd(0)** | **^176^Hf/^177^Hf** | **εHf(0)** | **δD_csw_** | **Match score** |
| East Central Europe | 0.69 | 0.31 | 0.717346 | 0.512101 | -10.32 | 0.282628 | -5.55 | -68.1 | 2 |
|  | 0.59 | 0.41 | 0.717485 | 0.512075 | -10.82 | 0.282650 | -4.79 | -69.7 | 1 |
|  | 0.86 | 0.14 | 0.718687 | 0.512070 | -10.92 | 0.282661 | -4.38 | -65.1 | 1 |
|  | 0.13 | 0.87 | 0.719259 | 0.512129 | -9.78 | 0.282648 | -4.85 | -74.1 | 2 |
|  | 0.44 | 0.56 | 0.719354 | 0.512085 | -10.63 | 0.282604 | -6.41 | -72.1 | 3 |
|  | 0.44 | 0.56 | 0.719422 | 0.512091 | -10.51 | 0.282623 | -5.71 | -71.6 | 3 |
|  | 0.57 | 0.43 | 0.719456 | 0.512079 | -10.75 | 0.282625 | -5.64 | -70.1 | 3 |
|  | 0.42 | 0.58 | 0.719475 | 0.512086 | -10.62 | 0.282625 | -5.66 | -70.8 | 3 |
|  | 0.40 | 0.60 | 0.719575 | 0.512100 | -10.34 | 0.282621 | -5.79 | -73.5 | 3 |
|  | 0.33 | 0.67 | 0.719643 | 0.512069 | -10.94 | 0.282601 | -6.52 | -74.2 | 3 |
|  | 0.17 | 0.83 | 0.719812 | 0.512111 | -10.12 | 0.282614 | -6.03 | -77.5 | 2 |
|  | 0.64 | 0.36 | 0.719835 | 0.512087 | -10.60 | 0.282631 | -5.45 | -66.4 | 2 |
|  | 0.25 | 0.75 | 0.719921 | 0.512096 | -10.41 | 0.282636 | -5.27 | -76.2 | 2 |
|  | 0.26 | 0.74 | 0.719992 | 0.512069 | -10.93 | 0.282609 | -6.23 | -75.3 | 2 |
|  | 0.62 | 0.38 | 0.720147 | 0.512075 | -10.82 | 0.282649 | -4.81 | -70.8 | 2 |
|  | 0.22 | 0.78 | 0.720197 | 0.512097 | -10.40 | 0.282625 | -5.66 | -76.2 | 2 |
|  | 0.95 | 0.05 | 0.720299 | 0.512070 | -10.93 | 0.282625 | -5.67 | -65.9 | 2 |
|  | 0.63 | 0.37 | 0.720343 | 0.512100 | -10.34 | 0.282638 | -5.19 | -70.0 | 3 |
| Ice marginal, Western and Central Europe | 0.82 | 0.18 | 0.718491 | 0.512110 | -10.13 | 0.282645 | -4.97 | -64.4 | 1 |
|  | 0.69 | 0.31 | 0.719264 | 0.512077 | -10.79 | 0.282576 | -7.39 | -65.7 | 2 |
|  | 0.62 | 0.38 | 0.719956 | 0.512079 | -10.76 | 0.282604 | -6.39 | -66.5 | 2 |
|  | 0.42 | 0.58 | 0.719973 | 0.512074 | -10.85 | 0.282574 | -7.46 | -69.0 | 2 |
|  |  |  |  |  |  |  |  |  |  |
| Mean of LGM ice core dust (GISP2, GRIP) |  |  | 0.718820 | 0.512138 | -9.60 |  |  |  |  |
| Mean of LGM ice core dust (NGRIP) |  |  | 0.719945 | 0.512095 | -10.44 | 0.282621 | -5.80 | -71.3 |  |
| F_A_ and F_B-C_ are the random simulated end-member fractions in mixtures (F_A_ - North Africa, F_B_ - East Central Europe, F_C_ - Ice marginal, Western and Central Europe) | | | | | | | | | |
| Match score is estimated against last glacial Greenland ice core dust isotope compositions: 1 - low, 2 - fair, 3 - high | | | | | | | | |  |
| Filtering of the original MC simulation dataset (displayed in Figure S10) was done using the isotopic range | | | | | | | |  |  |
| of LGM ice core dust (GISP2, GRIP and NGRIP), considering measurement reproducibilities: | | | | | | |  |  |  |
| δD_csw_: -80.3 to -62.3 permil |  |  |  |  |  |  |  |  |  |
| εHf(0): -7.57 to -4.16 |  |  |  |  |  |  |  |  |  |
| εNd(0): -10.97 to -8.74 |  |  |  |  |  |  |  |  |  |
| ^87^Sr/^86^Sr: 0.720387 to 0.717330 |  |  |  |  |  |  |  |  |  |


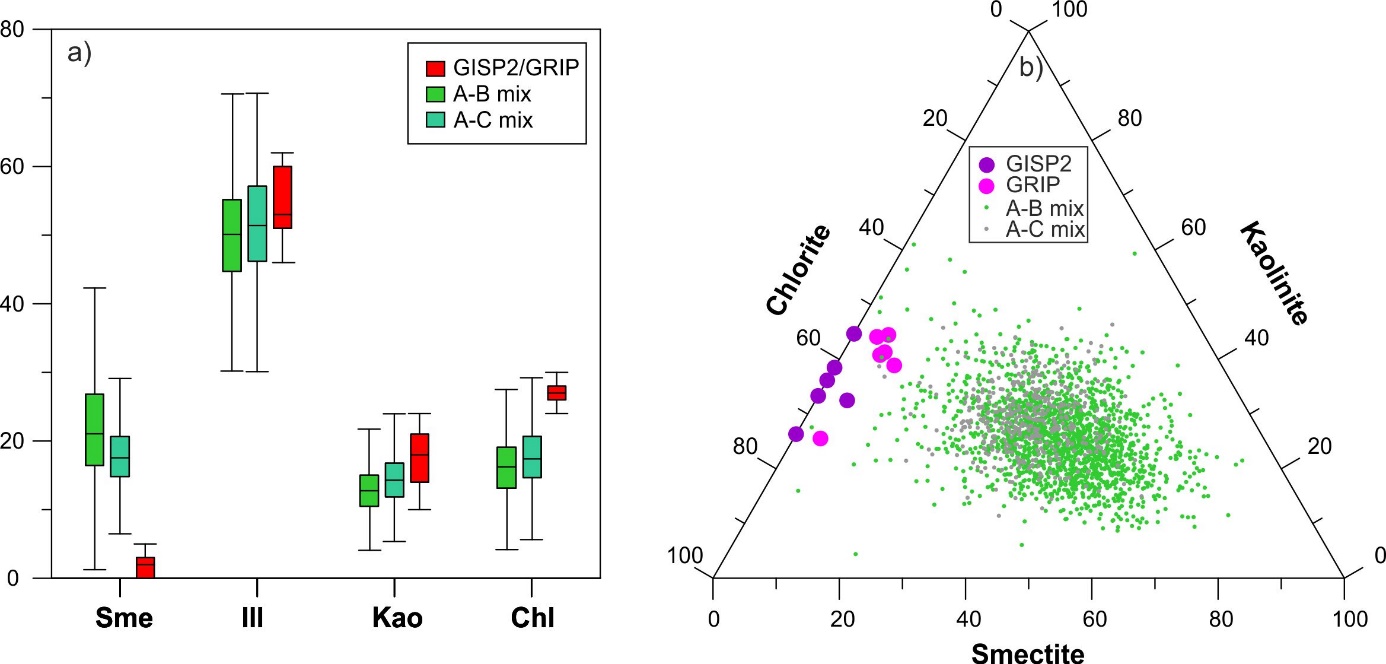


**Figure S15.** Box-whisker a) and ternary b) diagrams of simulated clay mineralogy of two component North African (A) and East Central (B) and Western European (C) dust mixtures using F_A_ and F_B-C_ end-member fractions given in Table S8. GISP2 and GRIP ice core dust compositions are also shown for comparison. Random sampling of clay mineralogy distributions was undertaken in 100 MC iterations for each F_A_ and F_B-C_ pairs given in Table S8 and thus simulation data displayed here include 2200 data points. The clay mineralogy distributions were defined from the XRD dataset given in Supporting Dataset S1 and were as follows: North African dust – smectite: 17±11 (mean±2SD), illite: 53±19, kaolinite: 15±8.6, chlorite: 16±11; East Central Europe - smectite: 26.1±26, illite: 47±19.4, kaolinite: 10.6±8, chlorite: 16.3±12; Ice marginal, Western ad Central Europe - smectite: 18.3±16, illite: 49±24, kaolinite: 13±9, chlorite: 20±7. More information in the main text.

**Text S8. Zircon depletion effects**

The Zr and Hf concentration data displayed in Figure S16 was obtained using an Agilent 7900 quadrupole ICP-MS at the Department of Environmental Geosciences, University of Vienna, Austria. The Nd isotope analyses were performed using a TIMS at the University of Vienna and the Hf isotope data using an MC-ICPMS at the Institute of Nuclear Research, Debrecen, Hungary (see main text, Methods). Aerosols were generated by resuspending loess/soil/red clay samples in an atmospheric chamber at the Laboratoire Interuniversitaire des Systèmes Atmosphériques (LISA), France. For further details see Supporting Dataset S4.

The decreasing Zr and Hf concentrations and more radiogenic Hf isotope compositions towards the finer fractions reflect the depletion of zircons in these aeolian deposits. There is an observable increase in εNd(0) values towards the finer fractions, which are limited to ~1 εNd unit. The Nd-Hf isotope compositions of the chamber generated aerosols match well those of the 8-16 μm fractions.


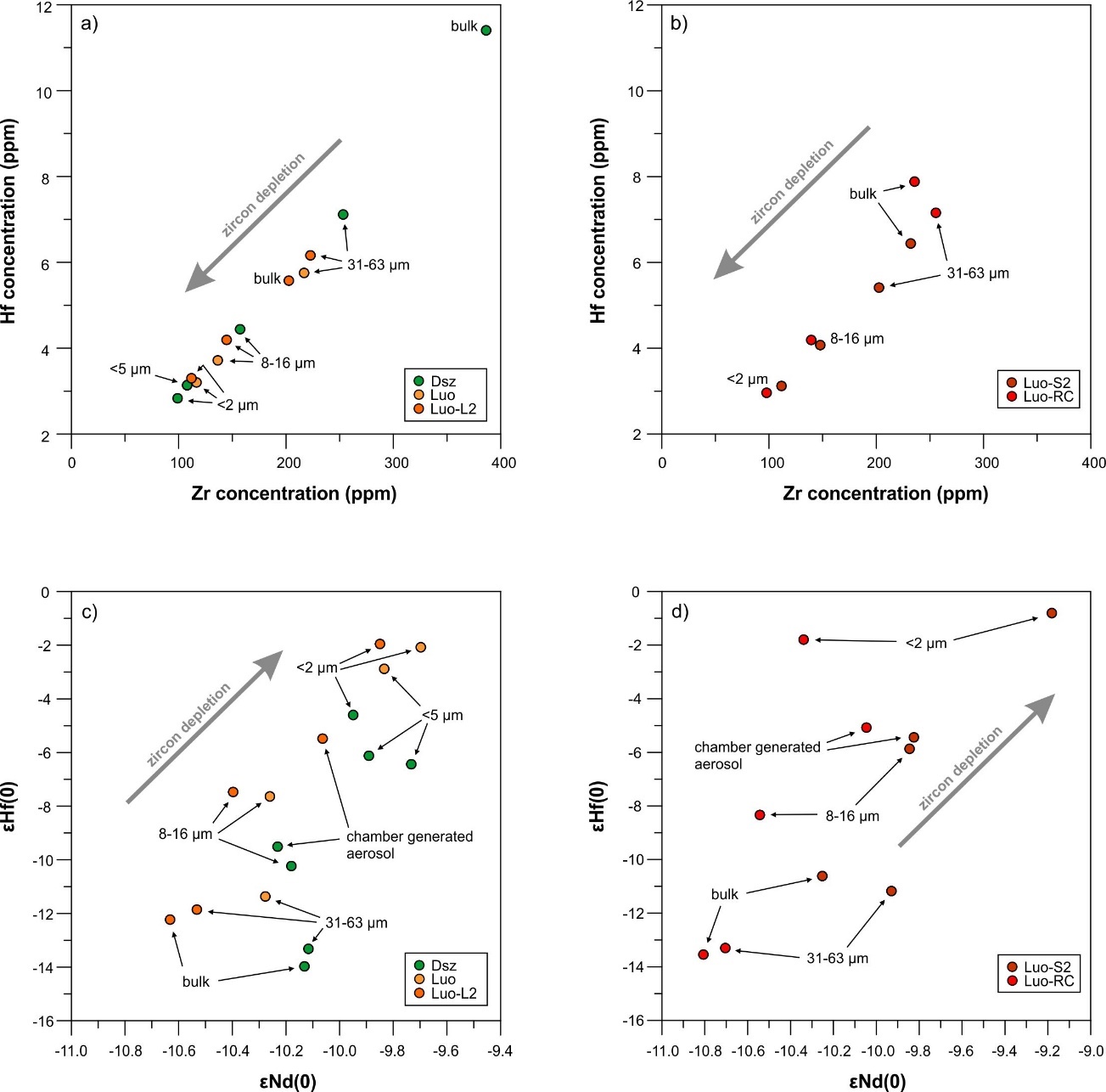


**Figure S16.** Zr and Hf concentrations (a,b) and Nd-Hf isotope compositions (c,d) of the bulk and various grain size fractions of aeolian loess, paleosol and red clays in Europe and SE Asia. Samples as listed in Supporting Dataset S4: Dsz – loess from the Dunaszekcső profile (Hungary), Luo – L1 loess from the Luochuan loess-paleosol-red clay sequence (China), Luo-L2 – L2 loess from the Luochuan sequence, Luo-S2 – S2 paleosol of the Luochuan record and Luo-RC – red clay from the Luochuan sequence.

**Text S9. Variations of Pb isotope ratios in various grain size fractions of aeolian deposits**

This note is based on data published by Feng et al. (2010), which demonstrate the grain size dependency of Pb isotope compositions of the aluminosilicate fractions of aeolian deposits. The original data and calculated Pb isotope ratios are listed in Table S9 and the absolute differences between the Pb isotope composition of the bulk sediment and seven isolated fractions are shown in Figure S17.

| **Table S9.** Pb isotope ratios of bulk and seven grain size fractions of aeolian deposits in China | | | | | | | | | | |  |  |  |
| --- | --- | --- | --- | --- | --- | --- | --- | --- | --- | --- | --- | --- | --- |
| **Sample^a^** | **Fraction (μm)^b^** | **^206^Pb/^204^Pb** | **2σ** | **^207^Pb/^204^Pb** | **2σ** | **^208^Pb/^204^Pb** | **2σ** | **^206^Pb/^207^Pb^c^** | **2σ** | **Δ^206^Pb/^207^Pb^d^** | **^208^Pb/^207^Pb^e^** | **2σ** | **Δ^208^Pb/^207^Pb^f^** |
| JTW1-3 | bulk silicate | 18.846 | 0.004 | 15.654 | 0.003 | 39.003 | 0.007 | 1.204 | 0.003 |  | 2.492 | 0.007 |  |
|  | >50 | 18.584 | 0.002 | 15.72 | 0.002 | 38.537 | 0.004 | 1.182 | 0.002 | 0.022 | 2.451 | 0.004 | 0.040 |
|  | 32-50 | 18.749 | 0.019 | 15.706 | 0.017 | 38.858 | 0.042 | 1.194 | 0.018 | 0.010 | 2.474 | 0.038 | 0.017 |
|  | 20-32 | 19.091 | 0.003 | 15.664 | 0.002 | 39.069 | 0.006 | 1.219 | 0.002 | 0.015 | 2.494 | 0.005 | 0.003 |
|  | 10-20 | 18.943 | 0.002 | 15.641 | 0.002 | 39.029 | 0.004 | 1.211 | 0.002 | 0.007 | 2.495 | 0.004 | 0.004 |
|  | 5-10 | 18.975 | 0.004 | 15.671 | 0.003 | 39.041 | 0.008 | 1.211 | 0.003 | 0.007 | 2.491 | 0.007 | 0.000 |
|  | 2-5 | 18.903 | 0.002 | 15.661 | 0.002 | 39.042 | 0.005 | 1.207 | 0.002 | 0.003 | 2.493 | 0.005 | 0.001 |
|  | <2 | 18.455 | 0.004 | 15.643 | 0.003 | 38.476 | 0.008 | 1.180 | 0.003 | 0.024 | 2.460 | 0.007 | 0.032 |
| GZ1-2 | bulk silicate | 19.157 | 0.004 | 15.695 | 0.003 | 39.338 | 0.008 | 1.221 | 0.003 |  | 2.506 | 0.007 |  |
|  | >50 | 18.945 | 0.007 | 15.683 | 0.007 | 39.107 | 0.018 | 1.208 | 0.007 | 0.013 | 2.494 | 0.016 | 0.013 |
|  | 32-50 | 18.928 | 0.008 | 15.67 | 0.007 | 39.031 | 0.017 | 1.208 | 0.007 | 0.013 | 2.491 | 0.016 | 0.016 |
|  | 20-32 | 19.034 | 0.003 | 15.69 | 0.002 | 39.231 | 0.006 | 1.213 | 0.002 | 0.007 | 2.500 | 0.005 | 0.006 |
|  | 10-20 | 19.005 | 0.011 | 15.673 | 0.009 | 39.190 | 0.022 | 1.213 | 0.010 | 0.008 | 2.500 | 0.020 | 0.006 |
|  | 5-10 | 18.694 | 0.002 | 15.649 | 0.002 | 38.834 | 0.005 | 1.195 | 0.002 | 0.026 | 2.482 | 0.005 | 0.025 |
|  | 2-5 | 18.663 | 0.03 | 15.667 | 0.025 | 38.799 | 0.061 | 1.191 | 0.027 | 0.029 | 2.476 | 0.055 | 0.030 |
|  | <2 | 18.999 | 0.018 | 15.692 | 0.016 | 38.710 | 0.039 | 1.211 | 0.017 | 0.010 | 2.467 | 0.035 | 0.040 |
| LH1-3 | bulk silicate | 18.958 | 0.004 | 15.653 | 0.003 | 38.973 | 0.009 | 1.211 | 0.003 |  | 2.490 | 0.007 |  |
|  | >50 | 19.66 | 0.001 | 15.67 | 0.001 | 38.654 | 0.003 | 1.255 | 0.001 | 0.043 | 2.467 | 0.002 | 0.023 |
|  | 32-50 | 18.929 | 0.006 | 15.658 | 0.005 | 38.949 | 0.001 | 1.209 | 0.005 | 0.002 | 2.487 | 0.008 | 0.002 |
|  | 20-32 | 19.015 | 0.003 | 15.657 | 0.003 | 38.996 | 0.008 | 1.214 | 0.003 | 0.003 | 2.491 | 0.007 | 0.001 |
|  | 10-20 | 18.665 | 0.002 | 15.592 | 0.002 | 38.573 | 0.005 | 1.197 | 0.002 | 0.014 | 2.474 | 0.005 | 0.016 |
|  | 5-10 | 18.963 | 0.003 | 15.639 | 0.002 | 39.030 | 0.006 | 1.213 | 0.002 | 0.001 | 2.496 | 0.005 | 0.006 |
|  | 2-5 | 18.719 | 0.003 | 15.625 | 0.003 | 38.620 | 0.008 | 1.198 | 0.003 | 0.013 | 2.472 | 0.007 | 0.018 |
|  | <2 | 19.499 | 0.004 | 15.707 | 0.003 | 38.703 | 0.008 | 1.241 | 0.003 | 0.030 | 2.464 | 0.007 | 0.026 |
| YJ-1 | bulk silicate | 18.973 | 0.002 | 15.726 | 0.002 | 39.419 | 0.004 | 1.206 | 0.002 |  | 2.507 | 0.004 |  |
|  | >50 | 18.912 | 0.003 | 15.725 | 0.002 | 39.318 | 0.005 | 1.203 | 0.002 | 0.004 | 2.500 | 0.004 | 0.006 |
|  | 32-50 | 18.891 | 0.002 | 15.698 | 0.002 | 39.107 | 0.004 | 1.203 | 0.002 | 0.003 | 2.491 | 0.004 | 0.015 |
|  | 20-32 | 18.95 | 0.002 | 15.714 | 0.002 | 39.368 | 0.004 | 1.206 | 0.002 | 0.001 | 2.505 | 0.004 | 0.001 |
|  | 10-20 | 18.641 | 0.003 | 15.634 | 0.003 | 38.759 | 0.006 | 1.192 | 0.003 | 0.014 | 2.479 | 0.006 | 0.027 |
|  | 5-10 | 19.539 | 0.005 | 15.738 | 0.004 | 38.824 | 0.009 | 1.242 | 0.004 | 0.035 | 2.467 | 0.008 | 0.040 |
|  | 2-5 | 18.889 | 0.003 | 15.678 | 0.003 | 38.998 | 0.007 | 1.205 | 0.003 | 0.002 | 2.487 | 0.007 | 0.019 |
|  | <2 | 18.684 | 0.004 | 15.65 | 0.004 | 38.645 | 0.009 | 1.194 | 0.004 | 0.013 | 2.469 | 0.009 | 0.037 |
| ^a^Samples: JTW1-3 - Chengdu Clay (Sichuan), GZ1-2 - Garze loess (Tibetan Plateau), LH1-3 - Linghui loess (Chinese Loess Plateau) and YJ-1 - Xigaze loess (Tibetan Plateau) | | | | | | | | | | | | | |
| ^b^All samples are HCl treated, aluminosilicate fractions | | | | |  |  |  |  |  |  |  |  |  |
| ^c^Calculated from measured ^206^Pb/^204^Pb and ^207^Pb/^204^Pb isotope ratios | | | | | |  |  |  |  |  |  |  |  |
| ^d^Absolute values of the difference between the ^206^Pb/^207^Pb isotope ratios of the bulk silicate and other grain size fractions | | | | | | | | | | |  |  |  |
| ^e^Calculated from measured ^207^Pb/^204^Pb and ^208^Pb/^204^Pb isotope ratios | | | | | |  |  |  |  |  |  |  |  |
| ^f^Absolute values of the difference between the ^208^Pb/^207^Pb isotope ratios of the bulk silicate and other grain size fractions | | | | | | | | | | |  |  |  |


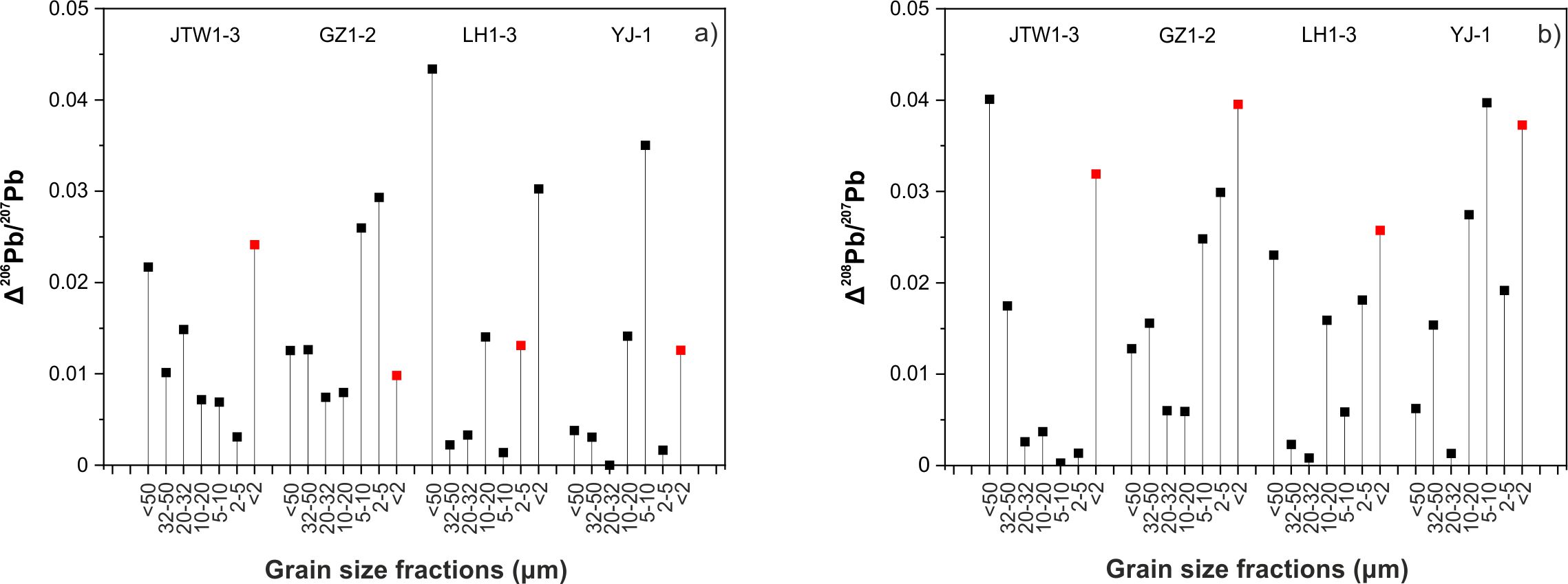


**Figure S17.** Absolute differences in a) ^206^Pb/^207^Pb and b) ^208^Pb/^207^Pb isotope ratios of seven grain size fractions as compared to the bulk of four aeolian deposits from China. Data source: Feng et al. (2010). Note the considerable variability in Pb isotope compositions of the bulk sediment and various grain size fractions. Red squares denote the clay (<2 μm) fraction usually considered in dust provenance discriminations of ice core dust.
